# Representation of Semantic Similarity in the Left Intraparietal Sulcus: Functional Magnetic Resonance Imaging Evidence

**DOI:** 10.3389/fnhum.2017.00402

**Published:** 2017-08-04

**Authors:** Veerle Neyens, Rose Bruffaerts, Antonietta G. Liuzzi, Ioannis Kalfas, Ronald Peeters, Emmanuel Keuleers, Rufin Vogels, Simon De Deyne, Gert Storms, Patrick Dupont, Rik Vandenberghe

**Affiliations:** ^1^Laboratory for Cognitive Neurology, Department of Neurosciences, University of Leuven Leuven, Belgium; ^2^Neurology Department, University Hospitals Leuven Leuven, Belgium; ^3^Department of Psychology, Centre for Speech, Language, and the Brain, University of Cambridge Cambridge, United Kingdom; ^4^Laboratory of Neurophysiology, Department of Neurosciences, University of Leuven Leuven, Belgium; ^5^Radiology Department, University Hospitals Leuven Leuven, Belgium; ^6^Department of Communication and Information Sciences, Tilburg University Netherlands; ^7^Humanities and Social Sciences Group, Laboratory of Experimental Psychology, University of Leuven Leuven, Belgium; ^8^Computational Cognitive Science Laboratory, University of Adelaide Adelaide, SA, Australia

**Keywords:** semantic processing, intraparietal sulcus, multi-voxel pattern analysis, object identity, geon, representational similarity analysis

## Abstract

According to a recent study, semantic similarity between concrete entities correlates with the similarity of activity patterns in left middle IPS during category naming. We examined the replicability of this effect under passive viewing conditions, the potential role of visuoperceptual similarity, where the effect is situated compared to regions that have been previously implicated in visuospatial attention, and how it compares to effects of object identity and location. Forty-six subjects participated. Subjects passively viewed pictures from two categories, musical instruments and vehicles. Semantic similarity between entities was estimated based on a concept-feature matrix obtained in more than 1,000 subjects. Visuoperceptual similarity was modeled based on the HMAX model, the AlexNet deep convolutional learning model, and thirdly, based on subjective visuoperceptual similarity ratings. Among the IPS regions examined, only left middle IPS showed a semantic similarity effect. The effect was significant in hIP1, hIP2, and hIP3. Visuoperceptual similarity did not correlate with similarity of activity patterns in left middle IPS. The semantic similarity effect in left middle IPS was significantly stronger than in the right middle IPS and also stronger than in the left or right posterior IPS. The semantic similarity effect was similar to that seen in the angular gyrus. Object identity effects were much more widespread across nearly all parietal areas examined. Location effects were relatively specific for posterior IPS and area 7 bilaterally. To conclude, the current findings replicate the semantic similarity effect in left middle IPS under passive viewing conditions, and demonstrate its anatomical specificity within a cytoarchitectonic reference frame. We propose that the semantic similarity effect in left middle IPS reflects the transient uploading of semantic representations in working memory.

## 1. Introduction

Previous studies of spatially selective attention have highlighted the contribution of the intraparietal sulcus (IPS) to spatial processing. IPS0/1, located in the descending segment of IPS, has been implicated in spatial-attentional enhancement of stimuli occurring in the contralateral attended hemifield (Yantis et al., 2002; Silver et al., 2005; Vandenberghe et al., 2005; Jerde et al., 2012). The middle IPS has been implicated in the coding of an attentional priority map, i.e., the spatial distribution of attentional weights (Vandenberghe and Gillebert, 2009, 2013). In middle IPS response amplitude has an asymptotic relationship with the number of items retained in working memory (Todd and Marois, 2004; Gillebert et al., 2012) which is mainly driven by the number of locations rather than the number of objects (Harrison et al., 2010).

Recently, it has become clear that features and objects held in working memory are also represented in the activity patterns in middle IPS (Ester et al., 2015; Bettencourt and Xu, 2016a) as well as the identity of abstract shapes (Christophel et al., 2012). This challenges the spatially oriented view of middle IPS and suggests that the information contained in its response patterns may be far richer in content (for review see Freud et al., 2016). Even more surprisingly, Devereux et al. (2013) reported semantic similarity effects in left IPS during a category naming task for six different categories (animals, clothing, insects, tools, vegetables, and vehicles). The fMRI similarity matrix for written words correlated with that obtained for pictures which led the authors to conclude that left middle IPS is involved in modality-invariant targeted retrieval of task-relevant semantic feature information (Devereux et al., 2013). This is surprising as there is no evidence of semantic processing deficits following IPS lesions.

The primary purpose of the current experiment was to determine whether the semantic similarity effect in middle IPS is replicable and how its localization compares to that of the visuospatial attention effects found previously in middle and posterior IPS (Vandenberghe et al., 2005; Molenberghs et al., 2008; Gillebert et al., 2013). A publicly available cytoarchitectonic reference frame was used to define the middle IPS region (Choi et al., 2006; Scheperjans et al., 2008a,b; Gillebert et al., 2013). hIP1 lies anteriorly in the depth of the IPS and hIP2 in the lower bank of IPS (Choi et al., 2006). Both areas are connected with the pars triangularis through the superior longitudinal fascicle (Uddin et al., 2010) and hIP2 also with the posterior part of the middle temporal gyrus (Uddin et al., 2010), hubs in the language and associative-semantic network (Vandenberghe et al., 2013; Liuzzi et al., 2017). hIP3 lies posteriorly and is connected with extrastriate cortex through the inferior fronto-occipital fascicle (Uddin et al., 2010). All three cytoarchitectonic areas are activated when subjects have to select between competing stimuli based on a prior spatial cue compared to single-grating trials, indicative of a role in spatially selective attention (Gillebert et al., 2013).

Effects in middle IPS (the sum of hIP1, hIP2, and hIP3) were directly compared to effects in posterior IPS. No cytoarchitectonic boundaries are available yet for posterior IPS and hence we defined this region based on coordinates derived from the contrast of contralateral vs. ipsilateral spatial attention in Vandenberghe et al. (2005). These coordinates are highly consistent between studies from different centers (Yantis et al., 2002; Gillebert et al., 2011) and correspond to what has been termed inferior IPS (Xu and Chun, 2006; Jeong and Xu, 2016) and IPS0/1 (Silver and Kastner, 2009). Semantic similarity effects in middle IPS were also compared to superior and inferior parietal regions. One of these regions, the left angular gyrus, has been classically implicated in semantic processing by univariate (for review see Binder et al., 2009) and multivariate pattern analyses (Bonner et al., 2013), serving as a positive control in the current study. To further assess the sensitivity and specificity of the analyses carried out in IPS, the same analyses were also performed in the ventral stream. To define the ventral stream region, we pooled cytoarchitectonically defined FG1, FG2 (Caspers et al., 2013, 2014), FG3 and FG4 (Lorenz et al., 2017).

One of the main study objectives was also to test whether an apparent semantic similarity effect in left middle IPS could be explained by visuoperceptual similarities. When using pictures, semantic similarity and visuoperceptual similarity may covary. The visuoperceptual similarity structure of the study stimulus set was estimated in three, complementary ways: behavioral estimates of subjective perceptual similarity, a shallow Neural Network model based on ventral stream visuoperceptual processing characteristics (Riesenhuber and Poggio, 2000) and a Deep Convolutional Neural Network (DCN) model (Krizhevsky et al., 2012). The DCN has been trained on a publicly available set of 1.2 million images of 1,000 different classes of ImageNet (ILSVRC-2010) (Krizhevsky et al., 2012). It consists of eight learned layers: five convolutional layers followed by three fully connected layers (Krizhevsky et al., 2012). The early, convoluted layers are principally determined by low-level visuoperceptual similarity. The connected layers partly reflect the learned categorization, i.e., the categorical labels assigned to the pictures during supervised learning and derived from higher-order perceptual similarities.

In order to determine the degree of specificity for semantically meaningful content, we also included geons, i.e., artificial 3D shapes constructed based on a formal algorithm with no learned semantic associations (Kayaert et al., 2003). A set of geons has a well-defined similarity structure based on visuoperceptual similarity. Geons were included in the current study in order to determine whether similarity effects observed in left middle IPS were conditional on the semantic memory content of the stimuli presented.

To minimize any interference by explicit task demands, subjects had to fixate the central fixation point in the absence of a task. A priori, passive viewing may be thought to have lower sensitivity for semantic similarity effects than e.g., a task requiring explicit semantic retrieval such as the category naming task used by Devereux et al. (2013). It may be expected to detect only the most robust and consistent effects. The absence of a task has as its main advantage that any results obtained are not contingent on the specific task characteristics. The middle IPS is a hub of the multidemand network and its activity level is affected by a wide range of tasks (Duncan, 2010). A priori, the task performed may influence the outcome of a semantic similarity analysis, e.g., depending on semantic control demands or the semantic content that needs to be retrieved (Lambon-Ralph et al., 2017). Passive viewing avoids a contribution of the task factor, rendering interpretation in terms of stimulus representation more straightforward.

The stimuli were taken from a previous independent fMRI study of object identity and semantic similarity (Bruffaerts et al., 2013a). In that fMRI study (Bruffaerts et al., 2013a) three inanimate categories were used, tools, vehicles, and musical instruments, in common with two related patient lesion studies (Vandenbulcke et al., 2006; Bruffaerts et al., 2014). The fMRI stimulus set remained within the inanimate category because semantic similarity is estimated based on a feature applicability matrix. The features of the animate and the inanimate category are so widely divergent that collecting one feature applicability matrix for both animate and inanimate entities entails highly artificial and often nonsensical questions (De Deyne et al., 2008). We restricted the set to two rather than three inanimate subcategories, vehicles and musical instruments, since the similarity of the fMRI responses in the previous study (Bruffaerts et al., 2013a) was higher for these two subcategories than for tools. Furthermore, this allowed for more replications per entity (*n* = 12 instead of 8) which is beneficial for fMRI similarity analysis at a fine-grained item-by-item level (Bruffaerts et al., 2013a).

## 2. Materials and methods

### 2.1. Participants

Thirty-one healthy subjects participated in the first experiment where pictures of real objects were presented (19–27 years old, 7 men). This experiment will be referred to below as the “main experiment.” Fifteen other subjects participated in the experiment with geons (19–28 years old, two men). This experiment will be referred to below as the “geon experiment.” All subjects were native Dutch speakers and strictly right-handed as tested by the Oldfield Inventory (Oldfield, 1971). The volunteers were free of psychotropic or vasoactive medication and had no neurological or psychiatric history. All participants gave written informed consent in accordance with the Declaration of Helsinki. The Ethics Committee of the University Hospitals Leuven approved the experimental protocol.

### 2.2. Stimuli

#### 2.2.1. Real-life stimuli

**Figure 1:**
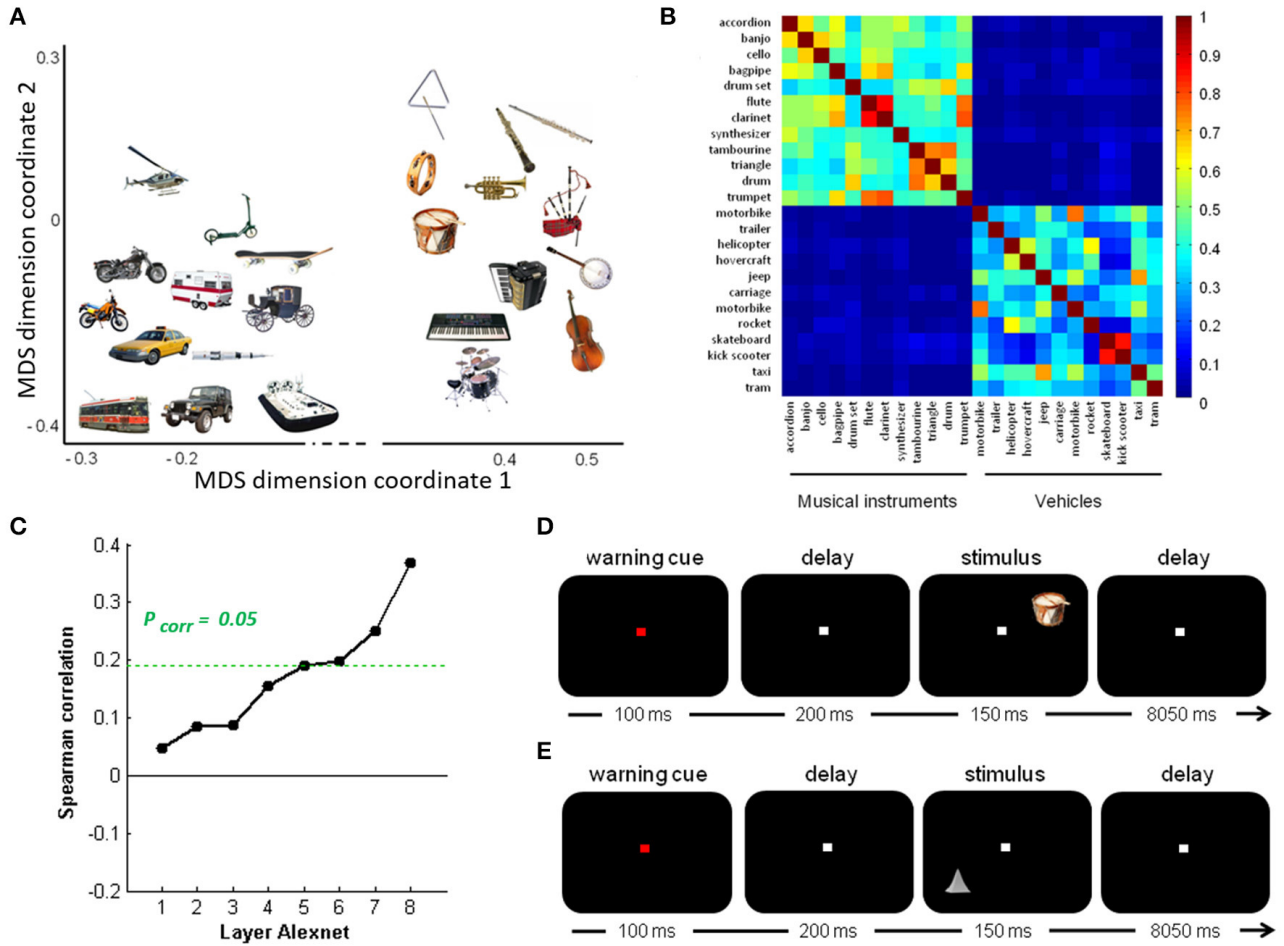
Overview of stimuli and design. **(A)** Semantic similarity between the real-life objects. Non-metric multidimensional scaling of the entities was performed based on an entity-by-entity dissimilarity matrix. To this end, the distance between each pair of rows in the concept-feature matrix was calculated with [1 − cosine similarity] as distance metric (De Deyne et al., 2008). The x and y values are in arbitrary units. **(B)** Semantic similarity matrix of the real-life stimuli. This matrix was derived from a feature generation task with subsequent scoring of the applicability of the most frequently generated features. **(C)** Deep learning. Spearman correlation between the semantic similarity matrix and the similarity matrices for the pictures for the eight layers of Alexnet. Statistical significance was evaluated by 10,000 random permutation labelings (green dotted line). Statistical threshold of *P* < 0.05, corrected for the number of layers. **(D)** Stimulus presentation: Schematic of the stimulus presentation with a sample real-life object as used in the main experiment and **(E)** a sample geon as used in the geon experiment.

The real-life stimulus set consisted of pictures of 24 objects (12 musical instruments and 12 vehicles) identical to those used by Bruffaerts et al. (2013a), presented against a black background (Figure 1A). Below the term “object” will be used to refer to the visual representation regardless of its size-on-the-screen, location or orientation, and the term “entity” for the concept represented by that image. Musical instruments and vehicles were matched for word frequency, word generation frequency, age of acquisition of the noun, familiarity, imageability and word length (Bruffaerts et al., 2013a). For each entity, a prototypical color photograph was selected. Familiarity and visual complexity of the color pictures were matched between the musical instruments and the vehicles, according to the familiarity ratings of 38 and the complexity ratings of 33 other volunteers, respectively (Bruffaerts et al., 2013a). The height or width of the picture, whichever was largest, was set to 5°.

The semantic similarity matrix for these objects was derived from a concept-feature matrix collected by De Deyne et al. (2008) based on a feature generation task performed by 1,003 college students for 229 concrete entities (De Deyne et al., 2008). In that study (De Deyne et al., 2008), each student generated 10 features for 10 entities each. The task instructions emphasized that different types of features had to be generated (e.g., perceptual, functional). Next, four different subjects scored the applicability of the most frequently generated features (*n* = 764) for every entity-feature combination. They were instructed to judge, for every entity-feature pair, whether the feature characterized the entity or not and, accordingly, to assign a 1 or a 0. Note that given the relatively high concordance of the applicability judgments between raters (0.80) and the large number of features probed, increasing the number of subjects above 4 would have only very limited effect on the similarity estimates. The resulting entity-feature matrix was converted into a semantic similarity matrix by calculating the cosine similarity between each pair of rows (Figure 1B). This matrix represents the semantic similarity between each possible pair of entities (De Deyne et al., 2008).

To quantify the subjective visuoperceptual similarity between the pictures, 11 volunteers rated the similarity between each possible pair of objects following the procedure outlined by Op de Beeck et al. (2008). There was no correlation between the subjective perceptual similarity matrix and the semantic similarity matrix (ρ = −0.041, *P* = 0.613).

To characterize the visuoperceptual similarities of the pictures in a more formal mathematical manner, the Alexnet Deep Convolutional Neural Network (DCN) was applied to this stimulus set (Krizhevsky et al., 2012). The pictures of the 12 musical instruments and 12 vehicles and their mirror images that we used for fMRI, were provided as input to the Deep Network. The activity pattern in each of the 8 layers was extracted for each picture. For each pair of pictures the cosine similarity of the corresponding activity vectors was calculated per layer. This resulted in eight similarity matrices, one for each layer. The Spearman correlation between the semantic similarity from the concept-feature matrix and the similarity matrices for each of the layers was determined. The values on the diagonal and the lower triangle of the matrices were excluded. Statistical significance was assessed by means of random permutations and a threshold of *P* < 0.05, corrected for the number of layers (*n* = 8). From layer 6 onwards (i.e., the fully connected layers), the similarity correlated significantly with the semantic similarity derived from the concept-feature matrix (Table 1, Figure 1C).

**Table 1:** Deep learning.

	**Semantic similarity matrix**	
	**Spearman ρ**	***P*-value**
Alexnet layer 1	0.0480	0.1876
Alexnet layer 2	0.0861	0.0814
Alexnet layer 3	0.0872	0.0739
Alexnet layer 4	0.1557	0.0129
Alexnet layer 5	0.1897	0.0069
Alexnet layer 6	**0.1974**	0.0045
Alexnet layer 7	**0.2497**	0.0002
Alexnet layer 8	**0.3681**	<0.0001

*Spearman correlation between the similarity matrices for the pictures for each of the eight layers of Alexnet and the semantic similarity matrix derived from the concept-feature matrix which is based on a feature generation task. Statistical significance was evaluated by 10,000 random permutation labelings. We applied a threshold of P < 0.05, corrected for the number of layers. Significant correlations are marked in bold*.

As a third manner of characterizing the visuoperceptual similarities between the pictures, HMAX, a shallow Neural Network model based on higher ventral stream characteristics (Riesenhuber and Poggio, 2000), was applied to the stimulus set. We extracted the C1 response vectors for each grayscaled picture using the HMAX package of the Cortical Network Simulator framework. The C1 units model complex cells that incorporate a limited tolerance to location and to size differences in each orientation. The cosine similarity was calculated for each pair of concatenated C1 response vectors, resulting in the C1 HMAX similarity matrix. Location- and scale-invariant shape similarity was assessed by means of the C2 layer of the HMAX model. The S2 layer was trained on 720 images with stereotypical views of the 24 entities of the current study which were taken from Google Images (30 images per entity). These images were entirely independent of the test set. The C2 layer extracts the maximum of the S2 responses over positions and scales for each feature, resulting in a feature vector. After training we tested quality of feature extraction in the C2 vectors by classifying the 24 objects used in the experiment. Accuracy of this classification was 79.17%. Next, we calculated the cosine similarity between each pair of C2 response vectors, resulting in the C2 HMAX similarity matrix. The C1 nor C2 HMAX similarity matrix correlated with the semantic similarity matrix (*P* > 0.09).

To evaluate possible phonological effects during picture processing, a phonological similarity matrix was created for the picture names by means of phonological transcriptions derived from the Dutch version of the CELEX lexical database (Baayen et al., 1993). The phonological similarity between each pair of objects was calculated as [1 − Levenshtein distance] (Liuzzi et al., 2017). We calculated the Spearman correlation between the semantic and the phonological similarity matrix after exclusion of the values on the diagonal. No correlation was found (ρ = 0.0565, *P* = 0.1678).

#### 2.2.2. Geons

In order to evaluate whether effects seen in IPS for real-life objects were specific for objects with an obvious semantic memory content, we also evaluated the effects for artificial shapes, namely geons (Kayaert et al., 2003). Gray-level rendered images of 8 sets (“families”) of 3 geons were created in 3D Studio Max, version 2.5. Each family of geons consisted of a reference shape, a metric property variant (MP) and a non-accidental property variant (NAP) (Hummel and Biederman, 1992; Kayaert et al., 2003). The geons were comparable to the one-part geons used by Kayaert et al. (2003). In the geon experiment, stimuli could have three possible sizes, resp. 4 × 4°, 4.5 × 4.5°, and 5 × 5°. All our one-part geons shared the same medial axis structure (geon skeleton, Lescroart and Biederman, 2013). The family structure within the set of the 24 geons can be represented by the “geon structural similarity matrix” (Hummel and Biederman, 1992). Furthermore, a C1 HMAX similarity matrix was derived for the geons in the same way as it was derived for the real-life objects. There was a significant second-order correlation between the geon structural similarity matrix and the C1 HMAX similarity matrix (ρ = 0.433, *P* < 0.001).

Subjective visuoperceptual similarity was based on the ratings of 10 volunteers, analog to the subjective visuoperceptual similarity for real-life objects. There was a significant second-order correlation between the geon structural similarity matrix and the subjective perceptual similarity matrix (ρ = 0.359, *P* < 0.001).

### 2.3. Experimental design

Each of the two experiments consisted of 12 runs containing 24 trials. At the start of each trial, a central fixation point turned from white to red during 100 ms. After a delay of 200 ms, one out of 24 stimuli was displayed in one of the quadrants of the screen on the diagonal at 2.5° eccentricity for 150 ms, followed by an intertrial interval of 8,050 ms (Figures 1D,E). The location was varied between the four quadrants pseudorandomly and counterbalanced over runs. The subjects were instructed to passively view the stimuli while fixating. Central gaze fixation was monitored using infrared eye recording (Eyelink 1000, SR Research).

In the main experiment each entity was shown once per run (Figure 1D). In 17 out of the 31 subjects, in half of the replications the picture was mirrored around the vertical axis similarly to a previous study which tested the invariance of the representations for orientation along with size and location (Bruffaerts et al., 2013a), in the remaining 14 subjects no mirroring was applied so as to avoid interaction effects between the degree of inherent symmetry of the images and the mirroring. Location was counterbalanced over runs for each subject. In the geon experiment each geon was shown once per run (Figure 1E). Location and size were counterbalanced over runs for each subject.

### 2.4. MRI acquisition and preprocessing

Structural and functional images were acquired on a 3T Philips Achieva system (Best, The Netherlands) equipped with a 32-channel head coil. Structural imaging sequences consisted of a T1-weighted 3D turbo-field-echo sequence (repetition time = 9.6 ms, echo time = 4.6 ms, in-plane resolution = 0.97 mm, slice thickness = 1.2 mm). Functional images were obtained using T2^*^ echoplanar images comprising 36 transverse slices (repetition time = 2 s, echo time = 30 ms, voxel size 2.75 × 2.75 × 3.75 mm^3^, slice thickness = 3.75 mm, Sensitivity Encoding (SENSE) factor = 2), with the field of view (FOV) (220 × 220 × 135 mm^3^) covering the entire brain. Each run was preceded by 4 dummy scans to allow for saturation of the Blood Oxygenation Level Dependent (BOLD) signal.

Preprocessing was performed with Statistical Parametric Mapping 2008 (SPM8) (Welcome Trust Centre for Neuroimaging, London, UK). The fMRI images were spatially realigned, slice time corrected and coregistered with the anatomical T1 image. Next, fMRI data were warped into MNI space by means of the spatial normalization parameters obtained from segmentation of the anatomical image. A voxel size of 3 × 3 × 3 mm^3^ was applied. For the univariate analysis the images were smoothed with a kernel size of 8 × 8 × 8 mm^3^, for the multivariate analysis the normalized, unsmoothed images were used.

### 2.5. Univariate analysis

A General Linear Model (GLM) was created in SPM8 with 8 event types, i.e., musical instruments and vehicles at each of the four stimulus locations. Six motion regressors were added as covariates of no interest. We contrasted all trials with baseline to determine the general pattern of activity evoked by the experimental trials and also contrasted the trials with vehicles to those with musical instruments. To evaluate location effects, we contrasted for each of the four stimulus locations the trials in which the object appeared in that location with all other experimental trials. The threshold was set at a voxel-level Family-Wise Error whole-brain corrected *P* < 0.05.

### 2.6. Volumes of interest for multivariate pattern analysis

The volumes of interest were defined based on cytoarchitectonic probabilistic mapping if available or else based on prior fMRI studies of spatial attention (Gillebert et al., 2013). For each individual, the overlap between the VOI and the individual's gray matter map was determined and only voxels containing more than 50% gray matter were retained (Table 2).

**Table 2:** Mean coordinate and number of voxels (voxel size 3 × 3 × 3 mm^3^) for each VOI.

	**Left hemisphere**					**Right hemisphere**				
	**Coordinate**			**Number of voxels**		**Coordinate**			**Number of voxels**	
	**x**	**y**	**z**	**Average**	**S.D**.	**x**	**y**	**z**	**Average**	**S.D**.
Middle IPS (hIP1-3)	−37	−51	43	195.9	23.0	39	−48	45	171.9	19.8
Posterior IPS	−20	−86	30	129.4	12.6	16	−86	34	146.3	15.7
Area 5	−14	−45	61	241.0	37.7	11	−48	63	231.4	24.2
Area 7	−18	−65	56	350.9	59.1	20	−64	58	295.2	37.4
Supramarginal	−56	−38	33	487.5	26.0	58	−35	32	569.0	42.1
Angular	−46	−68	32	337.2	20.9	50	−64	30	436.8	28.5
Fusiform gyrus	−38	−53	−18	489.1	25.0	39	−52	−19	435.1	23.1

**Figure 2:**
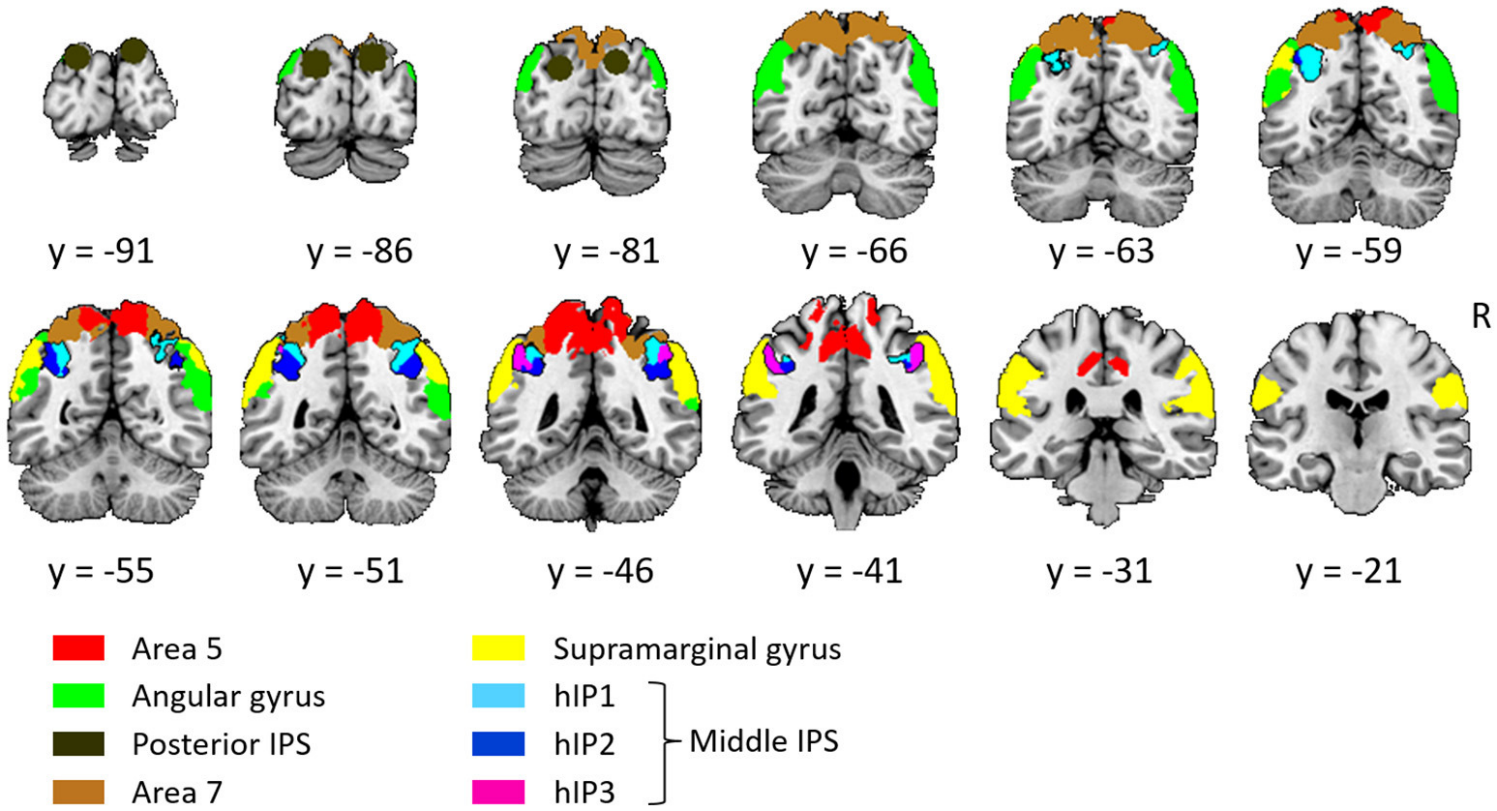
Overview of all volumes of interest. The primary focus was on middle IPS (sum of hIP1, hIP2, and hIP3), the other VOIs served as comparison. Except from posterior IPS, all VOIs were extracted from the Jülich-Düsseldorf cytoarchitectonic atlas by means of the Anatomy Toolbox (Eickhoff et al., 2005). The angular gyrus corresponds to the summation of area PGp and PGa; the supramarginal gyrus to area PFt, PFop, PFcm, PF, and PFm; area 5 to 5L, 5M, and 5Ci; area 7 to 7A, 7P, 7M, and 7PC. Posterior IPS VOIs was based on previously published fMRI MNI group coordinates (15, −87, 33 and −21, −87, 30; Vandenberghe et al., 2005).

*Coordinates are in MNI-space, average number of voxels and standard deviation per VOI are calculated over 46 subjects (31 subjects of the main experiment and 15 subjects of the geon experiment). Middle IPS refers to the sum of hIP1, hIP2, and hIP3; Supramarginal gyrus to the sum of area PFt, PFop, PFcm, PF, PFm (Caspers et al., 2006); Angular gyrus to the sum of area PGp and PGa (Caspers et al., 2006); Area 5 to the sum of area 5L, 5M, and 5Ci; Area 7 to the sum of area 7A, 7P, 7M, and 7PC (Scheperjans et al., 2005) (Figure 2); and Fusiform gyrus to the sum of posterior and middle fusiform cytoarchitectonic areas FG1, FG2 (Caspers et al., 2014), FG3 and FG4 (Lorenz et al., 2017)*.

The left and the right middle IPS volume of interest was obtained by summation of hIP1, hIP2 and hIP3 from the probabilistic brain atlas (Jülich-Düsseldorf cytoarchitectonic atlas) using the Anatomy Toolbox (Eickhoff et al., 2005) (Figures 2, **4A, 5A**). This combination of areas will be referred to below as hIP1-3. We verified the results in a functionally defined middle IPS volume based on a previous fMRI study (Gillebert et al., 2013). In that study, the contrast between trials with and without distractor resulted in an activation cluster in left and in right middle IPS. We created a sphere (12 mm radius) around the MNI peak coordinates (left: 33, 57, 48; right: 42, 42, 48) and the overlap between these VOIs and the individuals' gray matter was used for multivariate pattern analysis (MVPA).

Posterior IPS has not yet been characterized cytoarchitectonically. The posterior IPS VOI was defined bilaterally by creating a sphere (12 mm radius) centered on previously published fMRI MNI group coordinates (15, −87, 33 and −21, −87, 30, Vandenberghe et al., 2005) (Figures 2, **6A**).

For the sake of comparison, the cytoarchitectonic areas of the superior and inferior parietal cortex were also examined. In order to limit the number of statistical comparisons, area PFt, PFop, PFcm, PF, PFm were grouped as the supramarginal gyrus (Caspers et al., 2006), area PGp and PGa as the angular gyrus (Caspers et al., 2006), area 5L, 5M, and 5Ci as area 5, and area 7A, 7P, 7M, and 7PC as area 7 (Scheperjans et al., 2005) (Figure 2).

In order to verify the specificity and sensitivity of the analyses conducted in middle and posterior IPS, the same analyses were also performed in a ventral stream region, i.e., FG defined as the sum of posterior and middle fusiform cytoarchitectonic areas FG1, FG2 (Caspers et al., 2014), FG3 and FG4 (Lorenz et al., 2017), to the left and to the right.

Size differed between VOIs (Table 2) and this may theoretically affect the sensitivity of MVPA. For that reason we performed a secondary analysis where we equated the size between VOIs and evaluated whether we could replicate our findings. The size of the hIP1-3 VOI was made equal to that of the posterior IPS VOI by increasing the probability threshold for the hIP1, hIP2 and hIP3 maximum probability map (Eickhoff et al., 2005). This required a maximum probability threshold of 54% for left hIP1-3 [size 131.2 voxels (SD 16.1)] and 48% for right hIP1-3 [size 136.9 voxels (SD 15.3)]. For the VOIs outside IPS, the size was equated with that of the IPS VOIs by selecting only those constituent cytoarchitectonic areas that had a roughly comparable size (195 ± 60 voxels) to left hIP123. These areas were PF, PFm, PFt, PGa, PGp, 7A, FG3, and FG4.

### 2.7. Multivariate analysis

For MVPA, the fMRI time series was extracted from the unsmoothed images. Motion regressors and low-frequency trends were removed using SPM8. Next, for each of the 288 trials and for each voxel, the integral (the area under the curve) was calculated of the BOLD response between 2 and 8 s (Bruffaerts et al., 2013a; Liuzzi et al., 2017). Next, for each VOI and each trial, a vector was constructed, containing as many elements as the number of voxels within the VOI and per element this integral value of a given voxel. Next, the average vector was calculated per object per subject. This vector can be represented in a multidimensional space and will be referred to below as the individual's “response pattern” to a particular object in that VOI.

#### 2.7.1. Primary analysis: representational similarity analysis

A 24-by-24 entity-by-entity fMRI matrix was created per subject for each VOI. For every cell of the matrix, the cosine similarity was calculated between each pair of fMRI response patterns that corresponds to the entity pair of that cell. We excluded pairs of trials that contained stimuli presented at the same location in order to maximize the contribution of location-invariant object representations. Next, these individual fMRI similarity matrices were averaged per VOI over subjects. In each VOI, the Spearman correlation was calculated between the fMRI similarity matrix and the semantic similarity matrix for the entities (Kriegeskorte et al., 2008). The values on the diagonal and the lower triangle of the matrices were excluded (the matrix is symmetrical). To evaluate whether this second-order Spearman correlation differed from chance, it was compared to the values obtained based on 10,000 random permutation labelings. We used a one-tailed statistical threshold of *P* < 0.05 (Bruffaerts et al., 2013a).

Note that we first average the 24-by-24 fMRI cosine similarity matrix across subjects and then determine the cosine similarity with the semantic cosine similarity matrix (that is identical between subjects). In order to verify the normality of the distribution across subjects, we determined the distribution of the Spearman correlation coefficients between each individual's fMRI 24-by-24 cosine similarity matrix and the semantic cosine similarity matrix. We evaluated whether the distribution deviated from normality using the Shapiro-Wilk test and also whether the distribution differed from the null distribution (Student's *t*-test). Next, we computed for each individual a standardized position within the distribution. We calculated the *P*-value for the Spearman correlation coefficient between the individual's fMRI cosine similarity matrix and the semantic cosine similarity matrix (based on 10,000 random permutation labelings, see above). We transformed the *P*-value to a *Z* score and examined whether the distribution of the *Z* scores deviated from normality (Shapiro-Wilk) and whether the distribution differed from the null distribution.

If a semantic effect was found, we verified whether the effect differed between musical instruments and vehicles by means of a signed rank test (Bruffaerts et al., 2013a). To this end, we calculated for both musical instruments and vehicles in each subject the rank of the correlation within the subject-specific distribution generated by random permutation labeling over all stimuli. A two-tailed signed rank test was performed comparing the ranks of the same subject between the two categories. We applied a statistical threshold of *P* < 0.05.

We also compared subjects in whom mirroring of the objects was applied (*n* = 17) and those in whom it was not (*n* = 14). The individual's rank of the correlation was compared between both subject groups using a Mann-Whitney *U*-test.

Regions that demonstrated a semantic effect were further investigated by comparing the fMRI similarity matrix with the eight similarity matrices corresponding to the layers of the Alexnet Deep Neural Network. Each of these matrices were correlated with the fMRI similarity matrix of interest. As before, we included all pairs of trials except those that contained stimuli presented at the same location and only off-diagonal values were included in the analysis. Statistical significance was determined by means of 10,000 random permutation labelings. We applied a threshold of *P* < 0.05, corrected for the number of layers. The same procedure was used for the two HMAX similarity matrices, the subjective visuoperceptual similarity matrix and also for the phonological similarity matrix.

A same analysis was performed to examine the correlation between the fMRI response patterns evoked by geon stimuli and the corresponding geon structural similarity matrix (Figure 1D), the C1 HMAX similarity matrix and the subjective perceptual similarity matrix.

#### 2.7.2. Effect of object identity

In each VOI, the cosine similarity of the fMRI response pattern was calculated between each pair of trials in which a same entity was presented. Pairs of trials that had the stimulus location in common in addition to the entity were excluded to maximize the contribution of location-invariant object representations. Next, the cosine similarities were averaged over objects for each individual and then over individuals. To evaluate whether the cosine similarity differed from chance, 10,000 random permutations were performed. We used a one-tailed statistical threshold of *P* < 0.05 (Bruffaerts et al., 2013a).

If an effect was found, we repeated the analysis but confined the entities to either musical instruments or vehicles. Next, we calculated in each subject for both categories the rank of the result within the subject-specific distribution generated by random permutation labeling over all stimuli. A two-tailed signed rank test was performed comparing the ranks of the same subject between the two categories. We applied a statistical threshold of *P* < 0.05.

#### 2.7.3. Effect of location

In each VOI, the cosine similarity of the fMRI response patterns was calculated between each pair of trials that had the stimulus location in common. Trial pairs that had the entity in common in addition to the stimulus location were excluded from this analysis so as to minimize the contribution from object identity to the similarity measure. Next, these cosine similarity values were averaged for each individual and then averaged over subjects. This value was then compared to the probability distributions obtained with 10,000 random permutation labelings, excluding pairs of trials in which the same object was shown at a same location. We used a one-tailed statistical threshold of *P* < 0.05.

#### 2.7.4. Between-VOI comparison

When posterior or middle IPS demonstrated a significant effect, we compared it pairwise to the homotopical contralateral VOI and the other ipsilateral parietal VOIs by means of a one-tailed signed rank test (Bruffaerts et al., 2013b). For each subject in every VOI, the rank of the result was determined within the subject-specific distribution generated by random permutation labeling with a threshold of *P* < 0.05. Then, a signed rank test was performed comparing the ranks of the same subject between different VOIs.

## 3. Results

### 3.1. Univariate analysis

**Figure 3:**
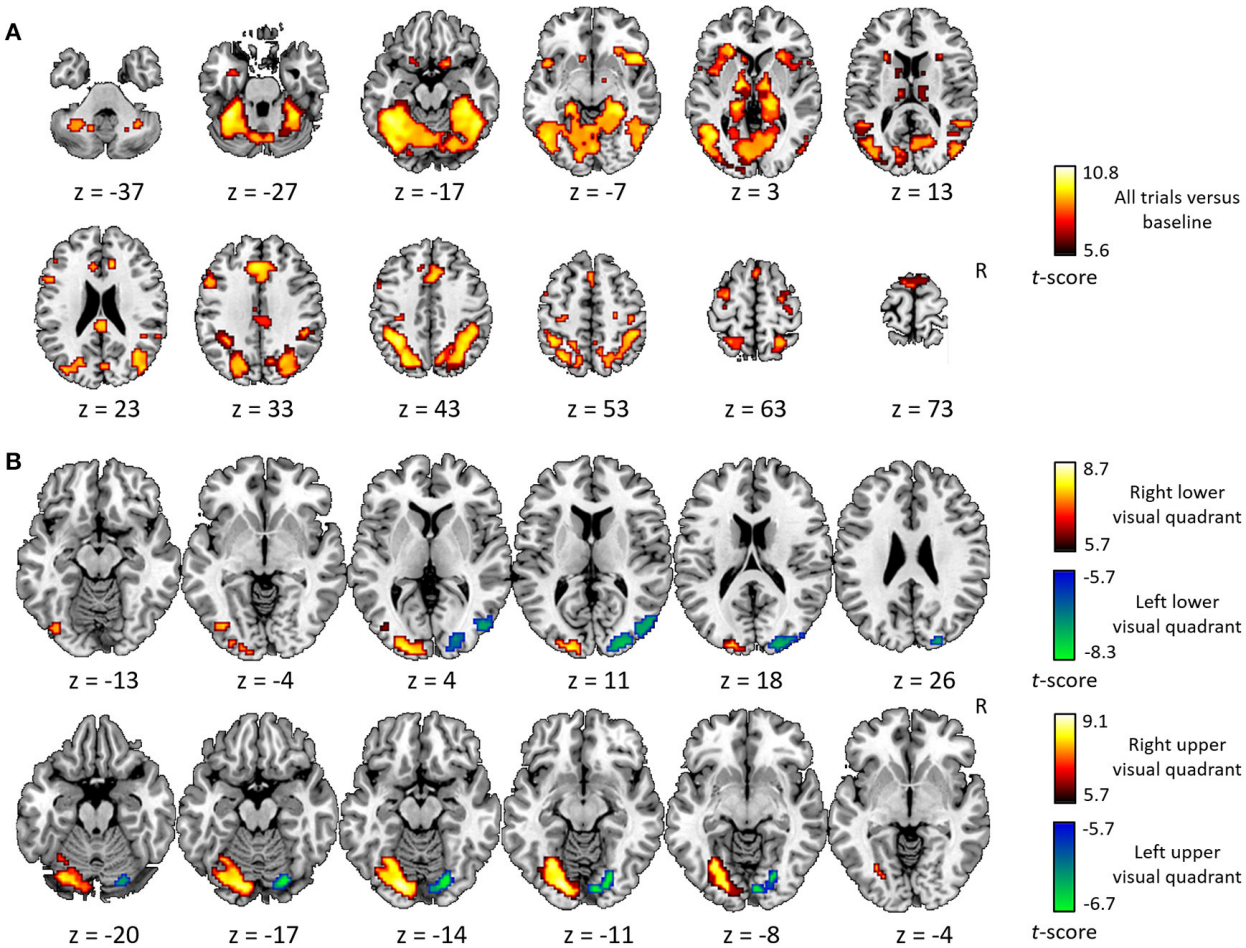
Univariate analysis. **(A)** Axial slices depicting the univariate contrast of all stimuli compared to baseline. **(B)** Axial slices depicting for each quadrant the univariate contrast with the three other quadrants. Results for the left visual field are displayed in green-blue and results for the right visual field in red-yellow, for the lower visual field (upper row) and the upper visual field (lower row). Voxel-level inference threshold of FWE whole-brain corrected *P* < 0.05.

**Figure 4:**
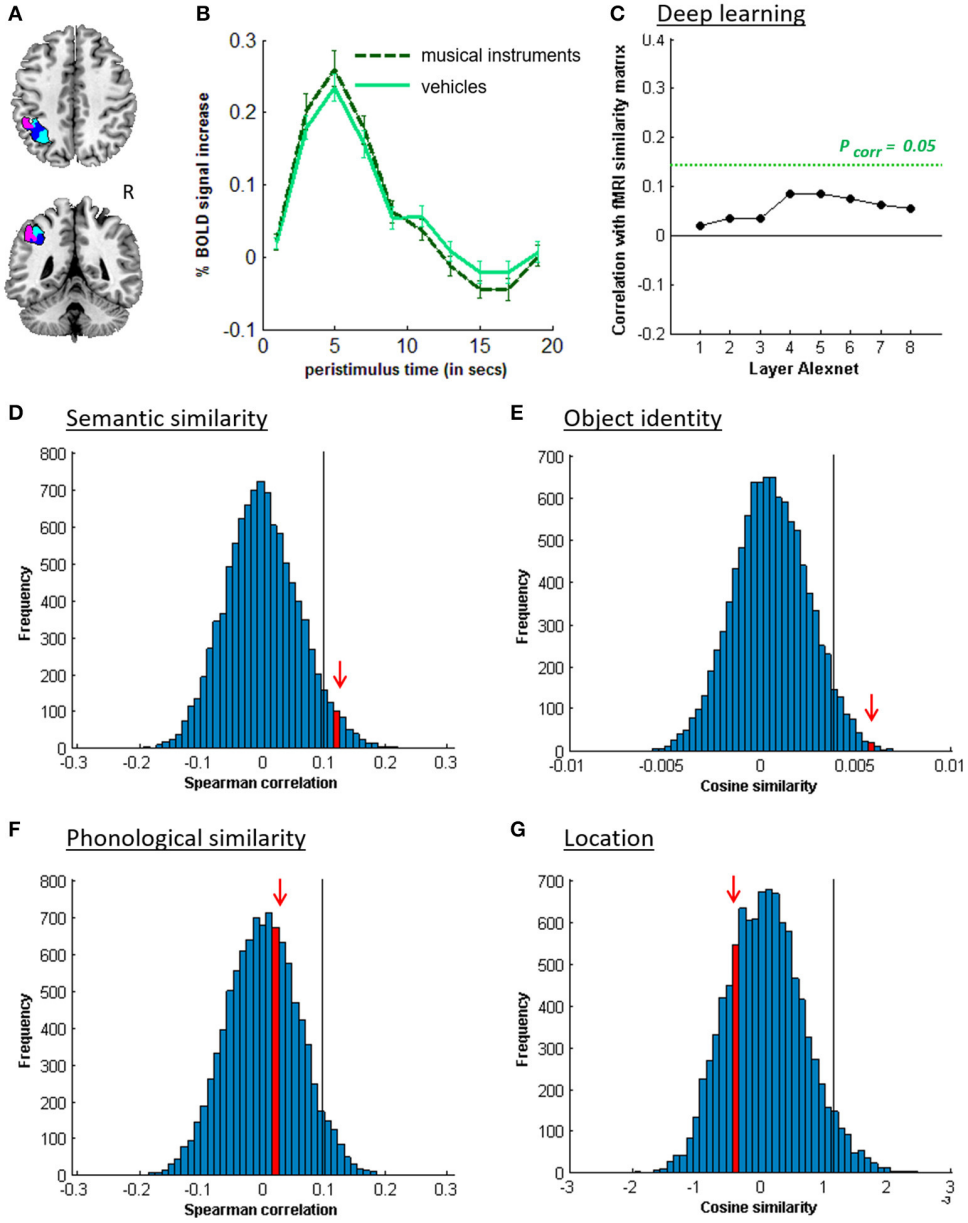
MVPA effects in left middle IPS (hIP1-3). **(A)** Axial (z = 43) and coronal (y = −46) slice depicting left middle IPS (hIP1, blue; hIP2, violet; hIP3; cyan). **(B)** Peristimulus respons for musical instruments and vehicles. **(C)** Deep Learning. Spearman correlation between the fMRI similarity matrix and the similarity matrices for the pictures for the eight layers of Alexnet. Statistical significance was evaluated by 10,000 random permutation labelings. The green dotted line corresponds to the statistical threshold corrected for the number of layers. **(D)** Probability distributions for the location-invariant semantic similarity effect. **(E)** Probability distributions for the location-invariant object identity effect. **(F)** Probability distributions for the phonological similarity effect. **(G)** Probability distributions for the object-invariant location effect. The red arrow indicates the true Spearman correlation/cosine similarity in the distribution of correlations/cosine similarities generated by 10,000 random permutations. X-axis: Spearman correlation averaged over the group of subjects. Y-axis: absolute frequency of a given average Spearman correlation value across all random permutation labelings. Black line: 95th percentile of the distribution.

**Figure 5:**
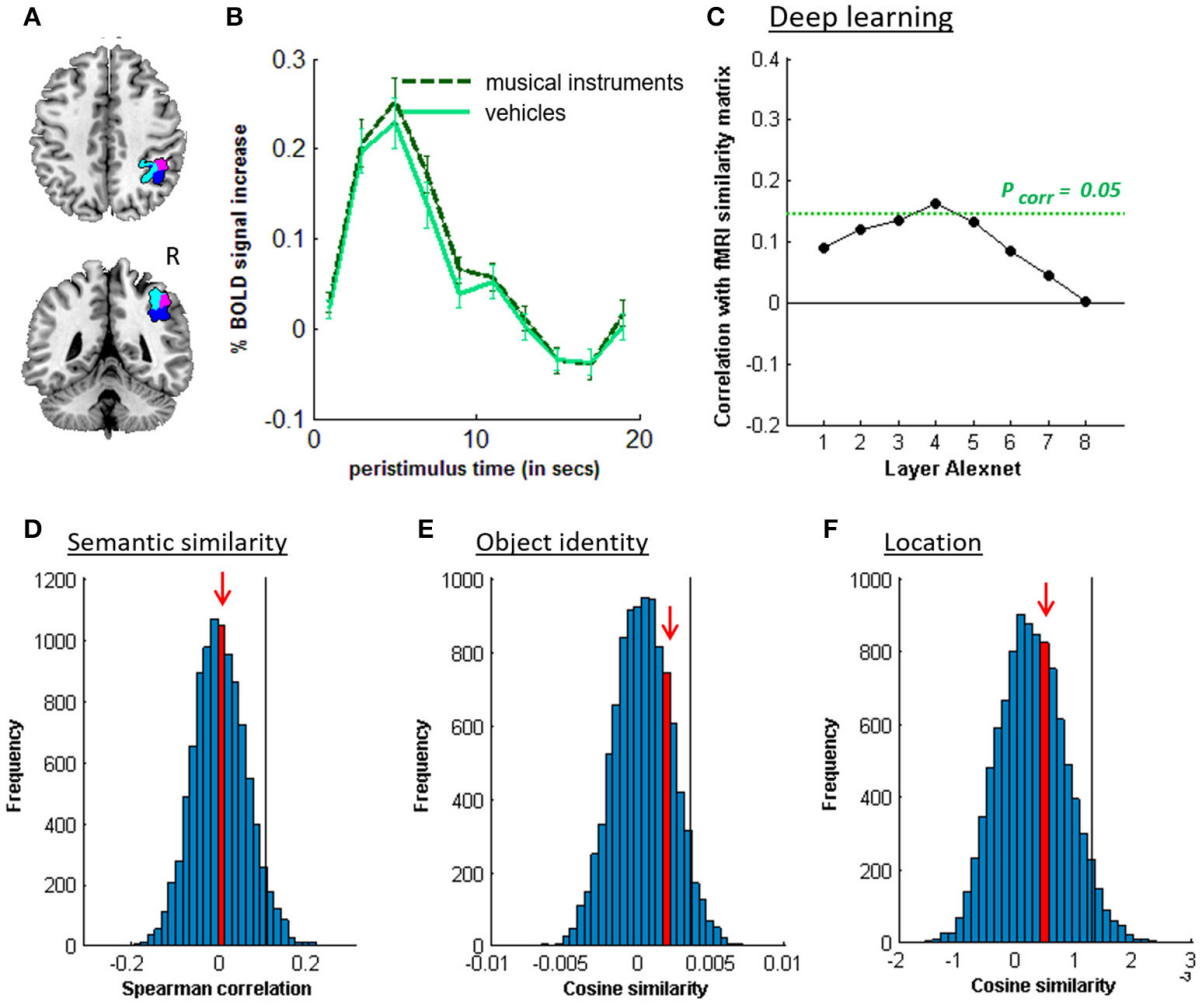
MVPA effects in right middle IPS (hIP1-3). **(A)** Axial (z = 43) and coronal (y = −46) slice depicting right middle IPS (hIP1, blue; hIP2, violet; hIP3, cyan). **(B)** Peristimulus response for musical instruments and vehicles. **(C)** Deep learning. Spearman correlation between the fMRI similarity matrix and the similarity matrices for the pictures for the eight layers of Alexnet. Statistical significance was evaluated by 10,000 random permutation labelings. The green dotted line corresponds to the statistical threshold corrected for the number of layers. **(D)** Probability distributions for the location-invariant semantic similarity effect. **(E)** Probability distributions for the location-invariant object identity effect. **(F)** Probability distributions for the object-invariant location effect. The red arrow indicates the true Spearman correlation/cosine similarity in the distribution of correlations/cosine similarities generated by 10,000 random permutations. X-axis: Spearman correlation averaged over the group of subjects. Y-axis: absolute frequency of a given average Spearman correlation value across all random permutation labelings. Black line: 95th percentile of the distribution.

**Figure 6:**
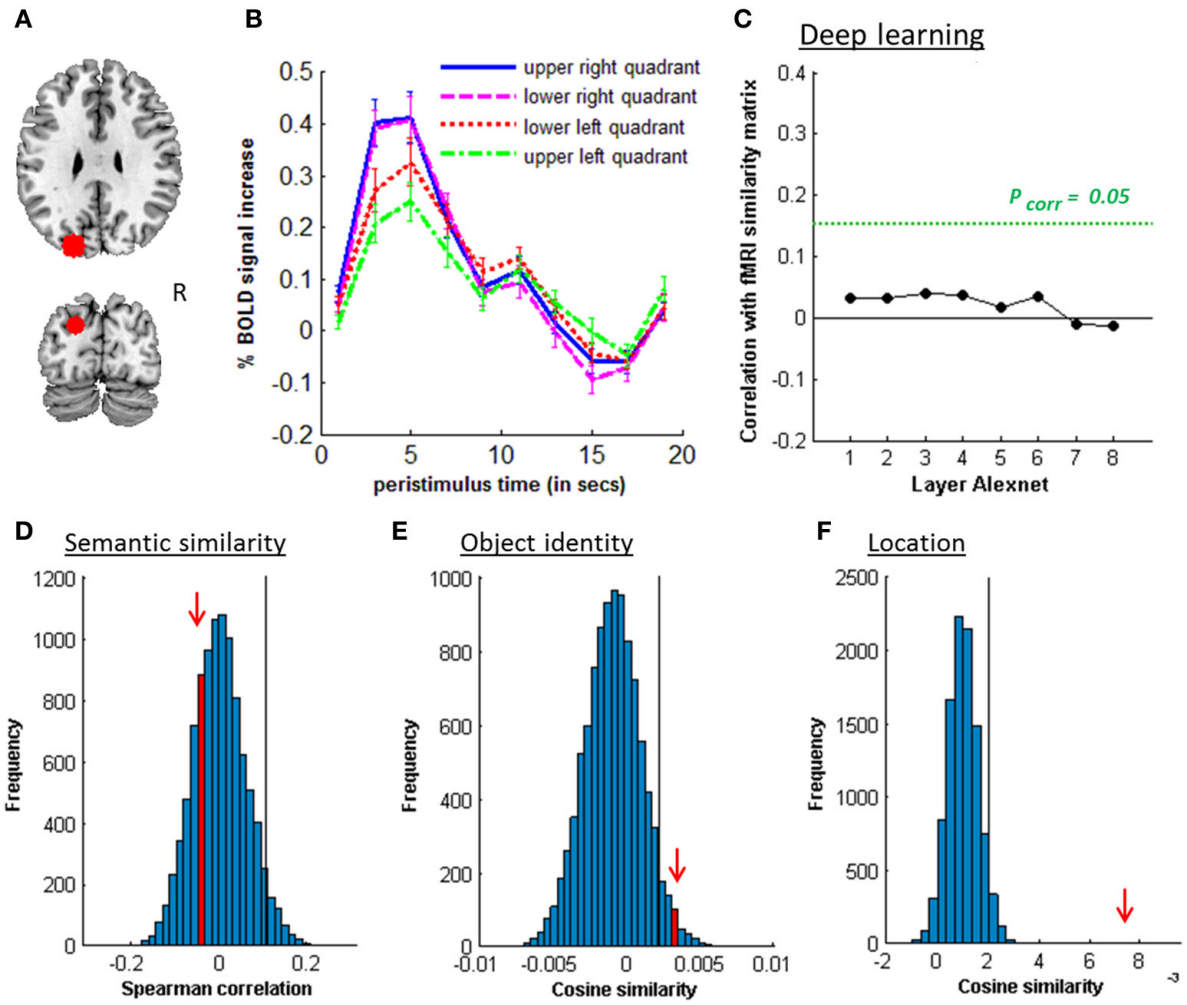
MVPA effects in left posterior IPS. **(A)** Axial (z = 30) and coronal (y = −80) slice depicting posterior IPS. **(B)** Peristimulus respons for each stimulus location. **(C)** Deep learning. Spearman correlation between the fMRI similarity matrix and the similarity matrices for the pictures for the eight layers of Alexnet. Statistical significance was evaluated by 10,000 random permutation labelings. The green dotted line corresponds to the statistical threshold corrected for the number of layers. **(D)** Probability distributions for the location-invariant semantic similarity effect. **(E)** Probability distributions for the location-invariant object identity effect. **(F)** Probability distributions for the location effect for real-life stimuli. The red arrow indicates the true Spearman correlation/cosine similarity in the distribution of correlations/cosine similarities generated by 10,000 random permutations. X-axis: Spearman correlation averaged over the group of subjects. Y-axis: absolute frequency of a given average Spearman correlation value across all random permutation labelings. Black line: 95th percentile of the distribution.

**Figure 7:**
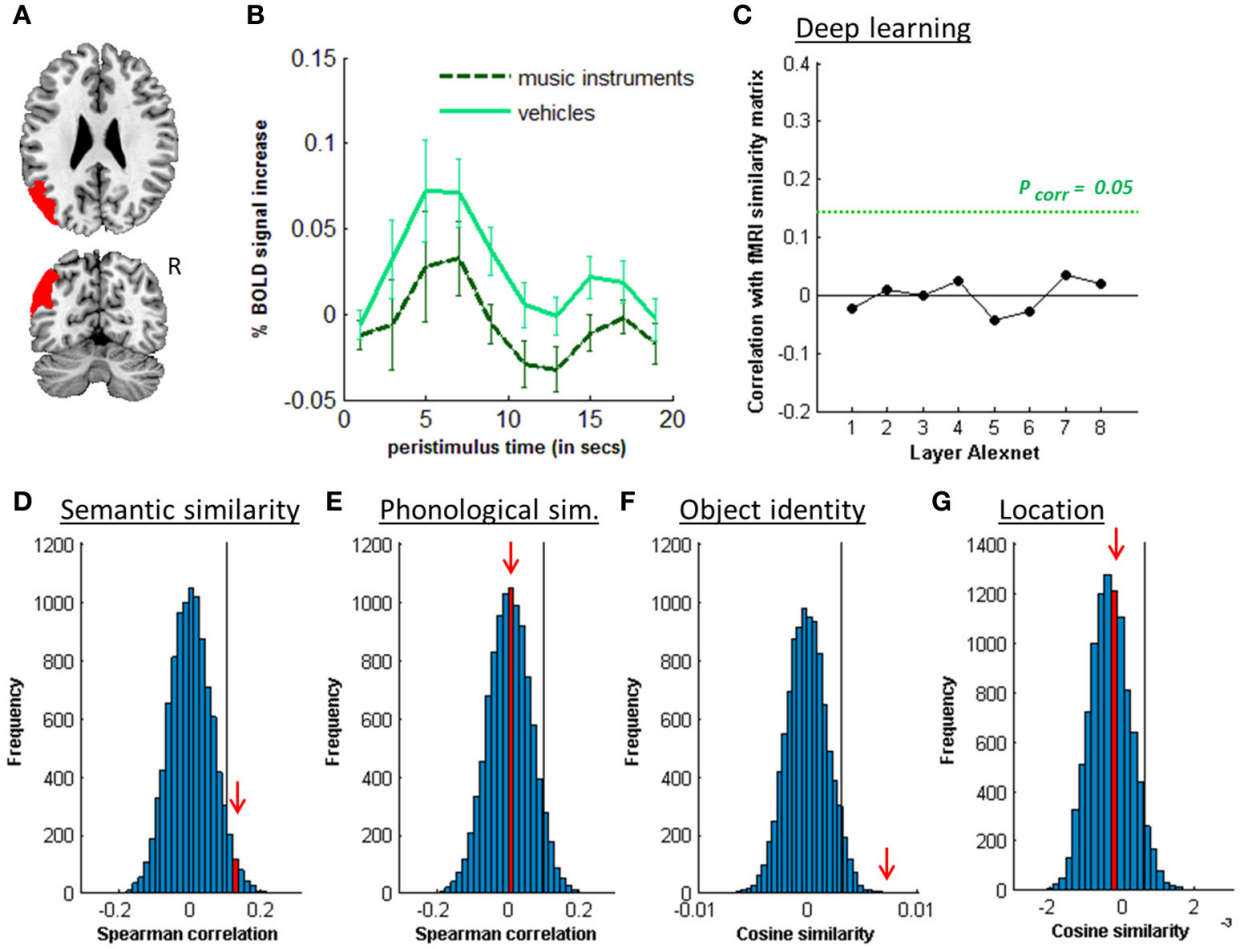
MVPA effects in the left angular gyrus. **(A)** Axial (z = 26) and coronal (y = −66) slice depicting posterior IPS. **(B)** Peristimulus response for musical instruments and vehicles. **(C)** Deep learning. Spearman correlation between the fMRI similarity matrix and the similarity matrices for the pictures for the eight layers of Alexnet. Statistical significance was evaluated by 10,000 random permutation labelings. The green dotted line corresponds to the statistical threshold corrected for the number of layers. **(D)** Probability distributions for the location-invariant semantic similarity effect. **(E)** Probability distributions for the phonological similarity effect. **(F)** Probability distributions for the location-invariant object identity effect. **(G)** Probability distributions for the object-invariant location effect. The red arrow indicates the true Spearman correlation/cosine similarity in the distribution of correlations/cosine similarities generated by 10,000 random permutations. X-axis: Spearman correlation averaged over the group of subjects. Y-axis: absolute frequency of a given average Spearman correlation value across all random permutation labelings. Black line: 95th percentile of the distribution.

The general activity pattern is shown in Figure 3A. The contrast between vehicles and musical instruments did not yield any significant differences (Figures 4B, 5B, 6B, 7B). Comparing each stimulus location with the remaining three locations resulted in an activity cluster for each location. The clusters of the lower quadrants were located lateral and superior in the occipital cortex (Figure 3B, upper row). For the upper quadrants, this led to inferior occipital activation of the contralateral hemisphere (Figure 3B, lower row). Peak coordinates and extent of each cluster can be found in Table 3. In a secondary analysis we looked for effects of stimulus location within the predefined VOIs using small volume correction. Significant effects were present in posterior IPS and in FG (Table 4).

**Table 3:** Cluster extent (voxel size 3 × 3 × 3 mm^3^), *P*-value, MNI peak coordinate and *Z*-value of the peak of the univariate activation for each stimulus location.

	**Cluster extent**	***P*-value cluster**	**Peak coordinate**	***Z*-value peak**
Upper left quadrant	63	<0.0001	22, −78, −16	5.20
Lower left quadrant	194	<0.0001	28, −93, 14	5.95
Upper right quadrant	268	<0.0001	−17, −81, −13	6.26
Lower right quadrant	154	<0.0001	−23, −96, 5	6.10
	45	<0.0001	−35, −78, −7	5.76

*The stimuli presented in one quadrant were contrasted with all other stimuli at a voxel-level inference threshold of FWE whole-brain corrected P < 0.05. For the lower right quadrant two clusters were found*.

**Table 4:** Univariate results with small volume correction.

	**Volume of interest**	**Peak coordinate**	***Z*-value peak**	***P***
Upper left quadrant	Right fusiform gyrus	34, −60, −19	4.45	0.002
Lower left quadrant	Right fusiform gyrus	43, −69, −10	4.92	0.000
	Right posterior IPS	25, −90, 26	5.29	0.000
Upper right quadrant	Left fusiform gyrus	−29, −66, −10	5.97	0.000
	Left posterior IPS	−26, −81, 23	4.63	0.000
Lower right quadrant	Left fusiform gyrus	−38, −75, −7	5.23	0.000
	Left posterior IPS	−20, −93, 20	5.67	0.000

*The first column mentions the first term of each contrast, the second (subtracted) term being all remaining conditions. For each contrast (Column 1), all VOIs with significant activation (Column 2) are displayed. Column 3–5: MNI peak coordinate, Z-value and voxel-level corrected P of the activation peak. The stimuli presented in one quadrant were contrasted with all other stimuli at a voxel-level treshold of corrected P < 0.05*.

### 3.2. Semantic similarity

Similarity between fMRI activity patterns in left hIP1-3 correlated significantly with semantic similarity between the objects presented (Table 5, Figure 4D). This effect was not present in right hIP1-3 (Figure 5D). The similarity effect was significantly stronger in left than in right hIP1-3 (*P* = 0.023). No semantic similarity effects were present in left or right posterior IPS (Table 5, Figure 6D). The semantic similarity effect in left hIP1-3 was significantly stronger than in left posterior IPS (*P* = 0.0335). Semantic similarity effects were present in hIP1, hIP2 as well as hIP3 when tested each separately (*P* < 0.039). The effect did not differ between musical instruments and vehicles (*P* = 0.442). The semantic similarity effect did not differ between subjects in whom mirroring of the images was applied and those in whom it was not (Mann Whitney *U*-test: *P* = 0.565).

**Table 5:** Main experiment.

	**Sem. similarity**		**Object identity**		**Location**	
	**Spearman ρ**	***P*-value**	**Cos. similarity**	***P*-value**	**Cos. similarity**	***P*-value**
L middle IPS (hIP1-3)	**0.1199**	0.0265	**0.0058**	0.0060	−0.0004	0.788
R middle IPS (hIP1-3)	0.0077	0.4393	0.0019	0.2087	0.0004	0.392
L posterior IPS	−0.0363	0.7164	**0.0033**	0.0150	**0.0077**	<0.0001
R posterior IPS	−0.0667	0.8667	**0.0012**	0.0407	**0.0085**	<0.0001

*Column 2–3: Spearman correlations between the semantic similarity matrix and the fMRI similarity matrix. Columne 4–7: cosine similarity (CS) for location and object identity. Significant results at an uncorrected P < 0.05 are marked in bold. L, left; R, right*.

When the size of hIP1-3 was equated to that of the posterior IPS VOI in a secondary analysis, results remained essentially the same (semantic similarity effect in the reduced L hIP1-3 ρ = 0.1070, *P* = 0.0388; in the reduced R hIP1-3, ρ = 0.0038, *P* = 0.5095). Direct comparison between IPS VOIs confirmed that the semantic effect in the reduced left hIP1-3 was significantly different from left posterior IPS (*P* = 0.0471) and from the reduced right hIP1-3 (*P* = 0.0489).

Analysis of the functionally defined middle IPS VOIs (Gillebert et al., 2013) confirmed these results. In the left middle IPS there was a significant semantic effect (ρ = 0.1206, *P* = 0.0305). In the right middle IPS no effect was found (ρ = 0.0670, *P* = 0.1359). The effect tended to be stronger in the left than in the right middle IPS (*P* = 0.078).

The analysis is based on the correlation between the 24-by-24 semantic similarity matrix and the 24-by-24 fMRI cosine similarity matrix averaged over subjects. Hence, it is important to evaluate the normality of the distribution of the correlations across subjects. The distribution of the Spearman correlation coefficients between the individuals' fMRI cosine similarity matrix in the left hIP1-3 and the semantic cosine similarity matrix did not significantly deviate from normality (Shapiro-Wilk test, *P* = 0.259). The distribution of the Spearman correlation coefficients significantly differed from the null distribution (Student's *t* = 1.853, *df* = 30, *P* = 0.037). Likewise, the distribution of the *Z* scores did not significantly deviate from normality (Shapiro-Wilk test, *P* = 0.979). The distribution of the *Z* scores significantly differed from the null distribution (Student's *t* = 1.823, *df* = 30, *P* = 0.037).

RSA based on the C1 and C2 HMAX similarity matrices did not reveal any effects in the left hIP1-3 (resp. *P* = 0.336 and *P* = 0.547). There were no significant correlations between the similarity of activity patterns in left hIP1-3 and any of the layers from the Alexnet Deep Learning model when corrected for the number of comparisons (*n* = 8), although there was a trend for layers 4–6 (Figure 4C). Neither was there an effect when the subjective visuoperceptual similarity matrix was used as input for RSA (ρ = −0.0533, *P* = 0.644). No effects of phonological similarity were found in left hIP1-3 (ρ = 0.0177, *P* = 0.376) (Figure 4F).

Outside the IPS, the left supramarginal gyrus and the left and right angular gyrus showed a significant semantic similarity effect (Table 6; Figure 7). The left supramarginal gyrus also demonstrated an effect of phonological similarity (ρ = 0.095, *P* = 0.043). In superior parietal area 5 and area 7, there were no semantic similarity effects (Table 6). When the size of the cytoarchitectonic areas outside IPS was matched to that of the left hIP1-3, semantic similarity effects were confirmed in cytoarchitectonic areas within left supramarginal and angular gyrus bilaterally, without effects in the superior parietal lobule (Table 7).

**Table 6:** Main experiment: Effects for real-life entities outside IPS.

	**Sem. similarity**		**Object identity**		**Location**	
	**Spearman ρ**	***P*-value**	**Cos. similarity**	***P*-value**	**Cos. similarity**	***P*-value**
L angular g.	**0.1206**	0.0254	**0.0067**	<0.0001	−0.0002	0.3810
R angular g.	**0.1333**	0.0161	**0.0040**	<0.0001	0.0012	0.08
L supramarginal g.	**0.0991**	0.0481	**0.0056**	<0.0001	−0.0006	0.3300
R supramarginal g.	0.0474	0.2107	**0.0037**	<0.0001	−0.0005	0.3660
L area 5	0.0591	0.1594	**0.0043**	0.0341	−0.0001	0.3403
R area 5	0.0923	0.0826	**0.0047**	0.007	0.0004	0.1
L area 7	0.0119	0.4150	**0.0034**	0.0189	0.0009	0.0831
R area 7	0.0001	0.4909	**0.0043**	0.01	**0.0026**	0.01
L FG	**0.1254**	0.0259	**0.0087**	<0.0001	**0.0046**	<0.0001
R FG	0.0717	0.1153	**0.0063**	0.001	**0.0056**	<0.0001

*Column 2–3: Spearman correlations between the semantic similarity matrix and the fMRI similarity matrix. Columns 4–7: cosine similarity (CS) for location and object identity. Significant results at an uncorrected P < 0.05 are marked in bold. Significant results at an uncorrected P < 0.05 are marked in bold. FG, fusiform gyrus, corresponding to the sum of cytoarchitectonic areas FG1, FG2, FG3, and FG4*.

**Table 7:** Control analysis for VOI size.

	**Left hemisphere**				**Right hemisphere**			
	**Number of voxels**		**Semantic similarity**		**Number of voxels**		**Semantic similarity**	
	**Average**	**S.D**.	**Spearman ρ**	***P*-value**	**Average**	**S.D**.	**Spearman ρ**	***P*-value**
PF	158.4	11.9	0.0820	0.0862	192.8	19.2	0.0287	0.3070
PFm	183.6	12.9	0.0451	0.2346	205.6	18.1	0.0555	0.1773
PFt	141.5	11.8	**0.1395**	0.0137	98.9	11.5	0.0052	0.4514
PGa	195.8	14.5	0.0247	0.3234	209.7	15.5	**0.1107**	0.0400
PGp	244.1	14.7	**0.1933**	0.0012	243.5	17.8	**0.1361**	0.0141
7A	236.4	24.1	0.0705	0.1285	133.3	18.8	0.0288	0.3139
FG3	146.1	11.1	0.0668	0.1298	118.1	9.5	0.0028	0.4574
FG4	185.9	10.9	**0.1558**	0.0087	147.2	11.4	**0.1383**	0.0152

*Semantic effect in restricted hIP123 area (probability treshold left 54%, right 48%) and in selected cytoarchitectonic areas. Column 1–2: Average number of voxels (voxel size 3 × 3 × 3 mm^3^) and standard deviation per area, calculated over 46 subjects (31 subjects of the real-life stimuli experiment and 15 subjects of the geon experiment). Column 3–4: Spearman correlations between the semantic similarity matrix and the fMRI similarity matrix. Significant results at an uncorrected P < 0.05 are marked in bold*.

In left FG, there was a significant semantic similarity effect (Table 6). This was of the same order of magnitude as that seen in left hIP1-3 (signed rank test: *P* = 0.64). There was also a significant correlation between fusiform fMRI patterns and the C2 layer of the HMAX model, both in the left (ρ = 0.136, *P* = 0.028) and in the right hemisphere (ρ = 0.146, *P* = 0.021). This effect of C2 HMAX in FG was significantly stronger than in left hIP1-3 (left FG vs. left hIP1-3: *P* = 0.0042). The similarity of the fMRI activity patterns in FG tended to correlate with the similarity of the responses in layers 3-6 of the AlexNet DCN in left FG (uncorr. *P* between 0.027 and 0.1). Subjective visuoperceptual similarity tended to correlate with similarity of activity patterns in FG (left FG: ρ = 0.1988, *P* = 0.0635; right FG: ρ = 0.178, *P* = 0.086). This did not differ significantly from left hIP1-3 (left FG vs. hIP1-3: *P* = 0.750, right FG vs. hIP1-3: *P* = 0.2194). In cytoarchitectonic areas within FG that were matched in size to left hIP1-3, left and right FG4 showed a significant semantic similarity effect (Table 7). There were also effects of visuoperceptual similarity. RSA with the C2 HMAX similarity matrix was significant in left and right FG3 and in the right FG4 (resp. *P* = 0.0091, *P* = 0.0028, *P* = 0.039). In left FG4 there was a significant correlation with AlexNet layer 4 (ρ = 0.171, *P* = 0.0061).

### 3.3. Object identity

The effect of object identity in left hIP1-3 was significant (Figure 4E, Table 5). An effect was also present in hIP1, hIP2, and hIP3 when tested separately (*P* < 0.014). The effect was present for musical instruments (average cosine similarity (CS) = 0.007, *P* = 0.012) and also for vehicles (CS = 0.005, *P* = 0.041) in left hIP1-3. An object identity effect was present in nearly all other parietal regions tested (except for the right hIP1-3) (Tables 5, 6; Figures 5E, 6E, 7F). In FG, the object identity effect was significant in both hemispheres (*P* < 0.001) (Table 6).

### 3.4. Location

There was a robust effect of location in left and right posterior IPS (Table 5; Figure 6F). There were no observable effects of location in hIP1-3 (Table 5; Figures 4G, 5F). The effect of location in left posterior IPS was significantly stronger than in left hIP1-3 and this was also true for the right posterior IPS compared to the right hIP1-3 (for both *P* < 0.001). In the right hemisphere a location effect was also found in area 7 (Table 6). Left and right fusiform gyrus exhibited a significant location effect as well (CS = 0.0046, *P* < 0.0001) (Table 6).

### 3.5. Geon experiment

For geons, there was an object identity effect in right hIP1-3 with a trend in the same direction in left hIP1-3 (Table 8). In posterior IPS, there was an object identity effect for geons on both sides as well as an effect of geon location (Table 8). Outside IPS, there was an object identity effect for geons in the angular and supramarginal gyrus bilaterally as well as area 7 bilaterally (*P* < 0.0025), with a further trend in right FG (*P* = 0.093). In the geon experiment, there was no significant correlation between any of the geon similarity matrices (geon structure, subjective visuoperceptual similarity, or C1 HMAX) and the similarity between activity patterns in any of the IPS VOIs (*P* > 0.14).

**Table 8:** Geon experiment: Cosine similarity (CS) for structural similarity, object identity and location.

	**Geon structural similarity**		**Object identity**		**Location**	
	**Spearman ρ**	***P*-value**	**Cos. similarity**	***P*-value**	**Cos. similarity**	***P*-value**
L middle IPS (hIP1-3)	0.0594	0.1571	0.0055	0.0807	0.0030	0.0690
R middle IPS (hIP1-3)	−0.0173	0.6139	**0.0083**	0.0042	0.0018	0.1200
L posterior IPS	−0.0770	0.9105	**0.0053**	0.0135	**0.0101**	<0.001
R posterior IPS	−0.1096	0.9617	**0.0056**	0.0037	**0.0085**	<0.001

*Significant results at an uncorrected P < 0.05 are marked in bold*.

## 4. Discussion

The current study replicates the semantic similarity effect Devereux et al. (2013) first described during category naming in left middle IPS, in the absence of a task. It localizes the effect within a cytoarchitectonic reference frame to hIP1, hIP2, and hIP3, and demonstrates that it is specific for left middle IPS compared to the right or to posterior IPS. In contrast, object identity effects were not anatomically specific at all: They were present in nearly all parietal regions tested. The semantic similarity effect in left middle IPS could not be explained by visuoperceptual similarities. The semantic similarity effect in left middle IPS was of a similar size as that of regions more classically associated with semantic processing such as the left angular gyrus or the anterior fusiform gyrus (FG4).

### 4.1. Effects of object identity and location

Effects of object identity were present across nearly all parietal areas. The relatively ubiquitous object identity effect is probably due to the fact that object identity effects can occur for a variety of reasons situated at different processing stages. Previous studies that showed an object identity effect in IPS were based on an active task, most typically a working memory (Christophel et al., 2012, 2015; Ester et al., 2015) or a semantic task (Jeong and Xu, 2016). The presence of object identity effects in IPS in the absence of an active task proves that coding of object identity by IPS is relatively spontaneous and does not critically require task-related working memory or task-related cognitive control.

Posterior IPS showed an effect of both location and identity (Tables 5, 8). Such a combined effect was also present in FG (Table 6), in accordance with previous reports (Schwarzlose et al., 2008; Carlson et al., 2011; Cichy et al., 2011, 2013; Bruffaerts et al., 2013a). We and others have previously hypothesized that the posterior IPS is involved in spatial attentional enhancement of perceptual units in the contralateral hemispace, prior to object identification (Xu and Chun, 2006). The current data suggest that location-invariant object identity is coded already at the level of posterior IPS.

The middle IPS did not show any measurable effects of location (Tables 5, 8). Retinotopic mapping studies in individual subjects have revealed a topographical organization of IPS up to the most anterior end (Silver et al., 2005; Swisher et al., 2007; Silver and Kastner, 2009). These topographic effects can be enhanced by increasing the degree of spatial attention (Saygin and Sereno, 2008). A previous study succeeded in decoding the memorized or attended location from response patterns in IPS2 (Jerde et al., 2012). It is possible that spatial effects would have emerged in middle IPS with increasing attentional or working memory demands of the task.

### 4.2. Effects of visuoperceptual similarity

When using pictures, visuoperceptual similarities may covary with semantic similarities. Hence, one of the study objectives was to evaluate the effect of visuoperceptual similarities on left middle IPS patterns in order to exclude that the apparent semantic similarity effect would be due to visuoperceptual similarity confounds (Bruffaerts et al., 2013a). A first step was to vary experimentally the location, size, and in some subjects, orientation of the images so as to maximize invariance of the semantic representations for visual surface features. Despite these variations a robust semantic similarity effect was found. A second step was to characterize the visuoperceptual similarities in multiple complementary ways. Both subjective ratings and two more formal mathematical ways of determining visuoperceptual similarity were used, HMAX and DCN (Figures 4C, 5C, 6C, 7C). These two models differ in fundamental ways from each other. The HMAX model is more exclusively based on visuoperceptual similarities while DCN originates from supervised learning using categorical labels and therefore contains also categorical effects derived from higher-order visuoperceptual similarities in its fully connected upper layers. None of the visuoperceptual similarity measures correlated with the similarity of the activity patterns in left middle IPS. For these different reasons, a visuoperceptual account of the semantic similarity effect in left middle IPS seems implausible. Left FG differed from left middle IPS in that it showed a clear effect of location (Tables 5, 6) and a correlation with similarity at the C2 HMAX level. Overall, this indicates that left FG is more influenced by visuoperceptual similarities than left middle IPS is.

The AlexNetwork has been built based on supervised learning according to categorical labels assigned to pictures and the connected layers partly reflect this learned categorization. The connected layers of the DCN correlated with the behavioral measures of semantic similarity (Figure 1C). In the fMRI dataset, in middle IPS there was a trend toward a correlation with AlexNet layers 5–8 (Figures 4C, 5C), and even more strongly so in FG for AlexNet layers 3–6. This is in line with prior evidence that the similarity between the connected layers of the AlexNet DCN correlates with the similarity of fMRI activity patterns in downstream regions of the ventral occipitotemporal pathway, e.g., area IT (Khaligh-Razavi and Kriegeskorte, 2014; Güçlü and van Gerven, 2015; Cichy et al., 2016) and also in anterior parts of the dorsal stream (Cichy et al., 2016).

### 4.3. Effects of semantic similarity

Originally, the effect of semantic similarity described in IPS by Devereux et al. (2013) was surprising as this region has been more typically implicated in spatial attention (for review see Vandenberghe and Gillebert, 2009) and spatial working memory (Todd and Marois, 2004). Furthermore, to the best of our knowledge, there is no evidence from patient lesion studies that left IPS lesions cause semantic processing deficits. There however is prior evidence from univariate functional imaging analyses that the left middle IPS is activated during associative-semantic tasks compared to visuoperceptual judgment tasks (Vandenberghe et al., 1996, 2013). The activation of IPS is often neglected in studies of semantic processing, despite its status as a connector hub within the associative-semantic network (Vandenberghe et al., 2013; Xu et al., 2016). According to the current data, this region is coding semantic content at a relatively fine-grained level. This is proof of a semantic representation and is not compatible with an explanation in terms of task difficulty or semantic control. Compared to left middle IPS, the left angular gyrus has received much more attention in the semantic memory literature (Vigneau et al., 2006; Binder et al., 2009; Bonner et al., 2013; Devereux et al., 2013; Seghier, 2013). In the current study, the left middle IPS functional profile (Figure 4) resembled that of the left angular gyrus (Figure 7).

The effect of semantic similarity in left middle IPS (Devereux et al., 2013) overlapped with the effects of spatially selective attention seen in hIP1, hIP2, and hIP3 in previous functional brain mapping studies (Vandenberghe et al., 2005; Gillebert et al., 2013; Schrooten et al., 2017). In hIP1, hIP2, and hIP3 the presence of competing distracters during a spatial cueing task causes activation compared to trials with single targets and no distracters (Gillebert et al., 2012; Schrooten et al., 2017). This can be explained by a working memory account as distracters may enter the working memory stage and hence lead to stronger activation in the case of double compared to single stimulation. A working memory account can also incorporate the current findings: It provides the flexibility for coding different types of objects (Jeong and Xu, 2016), both familiar objects and novel geons (Table 8). The generality of stimulus processing is incorporated in the notion of a “visual workspace” (Christophel et al., 2015) to which perceptual units gain access if their relevance is sufficiently high and also in the concept of a general-demand network of which IPS forms one of the principal hubs (Duncan, 2010).

Importantly, this generality of involvement does not preclude coding of entities at a high level of granularity, both in terms of identity and semantic similarity. The combination of generality (Wojciulik and Kanwisher, 1999; Duncan, 2010) and specificity makes it unlikely that the effects observed reflect representations that are stored at the middle IPS sites in a permanent manner. It fits better with a dynamic uploading of semantic representations depending on the circumstances. It is plausible that as a working memory region IPS can upload semantic memory data in a dynamic manner, just as it can upload grating orientations (Bettencourt and Xu, 2016b) or other data without obvious semantic content such as geons.

### 4.4. Potential study limitations

Methodologically, the fMRI similarity matrix was first averaged over subjects and then the correlation with the semantic similarity matrix was calculated and the significance determined. Given the fine-grained analysis at the level of pairs of individual objects and the limited number of replications of each object (*n* = 12), the correlation at the individual level is insufficiently reliable, hence the need to average over subjects. We ensured that the significant effects were not driven by outliers by assessing the distribution of the values in the fMRI similarity matrix across subjects, and also verified that the distribution of the values differed from the null hypothesis. Previous studies have provided clear evidence that the effects that reach significance using the method applied are highly replicable across different study cohorts (Bruffaerts et al., 2013a; Liuzzi et al., 2015) and between different centers (Clarke and Tyler, 2014).

The two categories we tested were based on a prior fMRI study (Bruffaerts et al., 2013a) but are of a special kind: double dissociations usually show that vehicles and musical instruments fall in between the divide between animate and inanimate (Capitani et al., 2003). The choice of one of the categories, musical instruments, may have contributed to the positive effect. The most anterior segment of IPS is implicated in grasping actions and IPS regions just posterior to the grasping area respond during naming of tools and graspable objects (Valyear et al., 2007). Bracci and Op de Beeck (2016) presented items from six categories (minerals, animals, fruits/vegetables, musical instruments, sports articles and tools) and found that the similarity of fMRI activity patterns in the dorsal stream reflected the action-related properties of items. In the current dataset, there was no significant difference in the semantic similarity or the object identity effect between the category of musical instruments and that of vehicles.

## Conclusion

Left middle IPS (hIP1, hIP2, hIP3) activity patterns represent semantic similarity between concrete entities in a robust manner. This is unexpected but has now been replicated across different centers and experimental conditions.

## Author contributions

VN: study design, data acquisition, data analysis and interpretation, drafting and revising manuscript, and final approval. RB: data analysis and interpretation, drafting and revising manuscript, and final approval. AL and IK: data analysis and interpretation, revising manuscript, and final approval. RP: data acquisition, data analysis, revising manuscript, and final approval. EK: data analysis and interpretation, revising manuscript, and final approval. RuV, GS, SD and PD: study design, data analysis and interpretation, revising manuscript, and final approval. RiV: study design, data analysis and interpretation, drafting and revising manuscript, and final approval.

### Conflict of interest statement

The authors declare that the research was conducted in the absence of any commercial or financial relationships that could be construed as a potential conflict of interest.

## References

[B1] Baayen H. Piepenbrock R. van Rijn H. (1993). The CELEX Lexical Database (CD-ROM). Philadelphia, PA: Linguistic Data Consortium.

[B2] Bettencourt K. C. Xu Y. (2016a). Decoding the content of visual short-term memory under distraction in occipital and parietal areas. Nat. Neurosci. 19, 150–157. 10.1038/nn.4174 26595654 PMC4696876

[B3] Bettencourt K. C. Xu Y. (2016b). Understanding location- and feature-based processing along the human intraparietal sulcus. J. Neurophysiol. 116, 1488–1497. 10.1152/jn.00404.2016 27440243 PMC5040374

[B4] Binder J. R. Desai R. H. Graves W. W. Conant L. L. (2009). Where is the semantic system? A critical review and meta-analysis of 120 functional neuroimaging studies. Cereb. Cortex 19, 2767–2796. 10.1093/cercor/bhp055 19329570 PMC2774390

[B5] Bonner M. F. Peelle J. E. Cook P. A. Grossman M. (2013). Heteromodal conceptual processing in the angular gyrus. Neuroimage 71C, 175–186. 10.1016/j.neuroimage.2013.01.006 PMC3594130 23333416

[B6] Bracci S. Op de Beeck H. (2016). Dissociations and associations between shape and category representations in the two visual pathways. J. Neurosci. 36, 432–444. 10.1523/JNEUROSCI.2314-15.2016 26758835 PMC6602035

[B7] Bruffaerts R. De Weer A.-S. De Grauwe S. Thys M. Dries E. Thijs V.. (2014). Noun and knowledge retrieval for biological and non-biological entities following right occipitotemporal lesions. Neuropsychologia 62, 163–174. 10.1016/j.neuropsychologia.2014.07.021 25080190

[B8] Bruffaerts R. Dupont P. De Grauwe S. Peeters R. De Deyne S. Storms G. (2013a). Right fusiform response patterns reflect visual object identity rather than semantic similarity. Neuroimage 83C, 87–97. 10.1016/j.neuroimage.2013.05.128 23811413

[B9] Bruffaerts R. Dupont P. Peeters R. De Deyne S. Storms G. Vandenberghe R. (2013b). Similarity of fMRI activity patterns in left perirhinal cortex reflects semantic similarity between words. J. Neurosci. 33, 18597–18607. 10.1523/JNEUROSCI.1548-13.2013 24259581 PMC6618797

[B10] Capitani E. Laiacona M. Mahon B. Caramazza A. (2003). What are the facts of semantic category-specific deficits? A critical review of the clinical evidence. Cogn. Neuropsychol. 20, 213–261. 10.1080/02643290244000266 20957571

[B11] Carlson T. Hogendoorn H. Fonteijn H. Verstraten F. A. J. (2011). Spatial coding and invariance in object-selective cortex. Cortex 47, 14–22. 10.1016/j.cortex.2009.08.015 19833329

[B12] Caspers J. Zilles K. Amunts K. Laird A. R. Fox P. T. Eickhoff S. B. (2014). Functional characterization and differential coactivation patterns of two cytoarchitectonic visual areas on the human posterior fusiform gyrus. Hum. Brain Mapp. 35, 2754–2767. 10.1002/hbm.22364 24038902 PMC4791049

[B13] Caspers J. Zilles K. Eickhoff S. B. Schleicher A. Mohlberg H. Amunts K. (2013). Cytoarchitectonical analysis and probabilistic mapping of two extrastriate areas of the human posterior fusiform gyrus. Brain Struct. Funct. 218, 511–526. 10.1007/s00429-012-0411-8 22488096 PMC3580145

[B14] Caspers S. Geyer S. Schleicher A. Mohlberg H. Amunts K. Zilles K. (2006). The human inferior parietal cortex: cytoarchitectonic parcellation and interindividual variability. Neuroimage 33, 430–448. 10.1016/j.neuroimage.2006.06.054 16949304

[B15] Choi H.-J. Zilles K. Mohlberg H. Schleicher A. Fink G. R. Armstrong E.. (2006). Cytoarchitectonic identification and probabilistic mapping of two distinct areas within the anterior ventral bank of the human intraparietal sulcus. J. Comp. Neurol. 495, 53–69. 10.1002/cne.20849 16432904 PMC3429851

[B16] Christophel T. B. Cichy R. M. Hebart M. N. Haynes J.-D. (2015). Parietal and early visual cortices encode working memory content across mental transformations. Neuroimage 106, 198–206. 10.1016/j.neuroimage.2014.11.018 25463456

[B17] Christophel T. B. Hebart M. N. Haynes J.-D. (2012). Decoding the contents of visual short-term memory from human visual and parietal cortex. J. Neurosci. 32, 12983–12989. 10.1523/JNEUROSCI.0184-12.2012 22993415 PMC6621473

[B18] Cichy R. M. Chen Y. Haynes J.-D. (2011). Encoding the identity and location of objects in human LOC. Neuroimage 54, 2297–2307. 10.1016/j.neuroimage.2010.09.044 20869451

[B19] Cichy R. M. Khosla A. Pantazis D. Torralba A. Oliva A. (2016). Comparison of deep neural networks to spatio-temporal cortical dynamics of human visual object recognition reveals hierarchical correspondence. Sci. Rep. 6:27755. 10.1038/srep27755 27282108 PMC4901271

[B20] Cichy R. M. Sterzer P. Heinzle J. Elliott L. T. Ramirez F. Haynes J.-D. (2013). Probing principles of large-scale object representation: category preference and location encoding. Hum. Brain Mapp. 34, 1636–1651. 10.1002/hbm.22020 22371355 PMC6870376

[B21] Clarke A. Tyler L. K. (2014). Object-specific semantic coding in human perirhinal cortex. J. Neurosci. 34, 4766–4775. 10.1523/JNEUROSCI.2828-13.2014 24695697 PMC6802719

[B22] De Deyne S. Verheyen S. Ameel E. Vanpaemel W. Dry M. J. Voorspoels W.. (2008). Exemplar by feature applicability matrices and other dutch normative data for semantic concepts. Behav. Res. Methods 40, 1030–1048. 10.3758/BRM.40.4.1030 19001394

[B23] Devereux B. J. Clarke A. Marouchos A. Tyler L. K. (2013). Representational similarity analysis reveals commonalities and differences in the semantic processing of words and objects. J. Neurosci. 33, 18906–18916. 10.1523/JNEUROSCI.3809-13.2013 24285896 PMC3852350

[B24] Duncan J. (2010). The multiple-demand (md) system of the primate brain: mental programs for intelligent behaviour. Trends Cogn. Sci. 14, 172–179. 10.1016/j.tics.2010.01.004 20171926

[B25] Eickhoff S. B. Stephan K. E. Mohlberg H. Grefkes C. Fink G. R. Amunts K.. (2005). A new spm toolbox for combining probabilistic cytoarchitectonic maps and functional imaging data. Neuroimage 25, 1325–1335. 10.1016/j.neuroimage.2004.12.034 15850749

[B26] Ester E. F. Sprague T. C. Serences J. T. (2015). Parietal and frontal cortex encode stimulus-specific mnemonic representations during visual working memory. Neuron 87, 893–905. 10.1016/j.neuron.2015.07.013 26257053 PMC4545683

[B27] Freud E. Plaut D. C. Behrmann M. (2016). ‘What’ is happening in the dorsal visual pathway. Trends Cogn. Sci. 20, 773–784. 10.1016/j.tics.2016.08.003 27615805

[B28] Gillebert C. R. Dyrholm M. Vangkilde S. Kyllingsbk S. Peeters R. Vandenberghe R. (2012). Attentional priorities and access to short-term memory: parietal interactions. Neuroimage 62, 1551–1562. 10.1016/j.neuroimage.2012.05.038 22634216

[B29] Gillebert C. R. Mantini D. Peeters R. Dupont P. Vandenberghe R. (2013). Cytoarchitectonic mapping of attentional selection and reorienting in parietal cortex. Neuroimage 67, 257–272. 10.1016/j.neuroimage.2012.11.026 23201362

[B30] Gillebert C. R. Mantini D. Thijs V. Sunaert S. Dupont P. Vandenberghe R. (2011). Lesion evidence for the critical role of the intraparietal sulcus in spatial attention. Brain 134(Pt 6), 1694–1709. 10.1093/brain/awr085 21576110

[B31] Güçlü U. van Gerven M. A. J. (2015). Deep neural networks reveal a gradient in the complexity of neural representations across the ventral stream. J. Neurosci. 35, 10005–10014. 10.1523/JNEUROSCI.5023-14.2015 26157000 PMC6605414

[B32] Harrison A. Jolicoeur P. Marois R. (2010). “What” and “Where” in the intraparietal sulcus: an fMRI study of object identity and location in visual short-term memory. Cereb. Cortex 20, 2478–2485. 10.1093/cercor/bhp314 20100899 PMC2936801

[B33] Hummel J. E. Biederman I. (1992). Dynamic binding in a neural network for shape recognition. Psychol. Rev. 99, 480–517. 10.1037/0033-295X.99.3.480 1502274

[B34] Jeong S. K. Xu Y. (2016). Behaviorally relevant abstract object identity representation in the human parietal cortex. J. Neurosci. 36, 1607–1619. 10.1523/JNEUROSCI.1016-15.2016 26843642 PMC4737772

[B35] Jerde T. A. Merriam E. P. Riggall A. C. Hedges J. H. Curtis C. E. (2012). Prioritized maps of space in human frontoparietal cortex. J. Neurosci. 32, 17382–17390. 10.1523/JNEUROSCI.3810-12.2012 23197729 PMC3544526

[B36] Kayaert G. Biederman I. Vogels R. (2003). Shape tuning in Macaque inferior temporal cortex. J. Neurosci. 23, 3016–3027. 12684489 10.1523/JNEUROSCI.23-07-03016.2003 PMC6742095

[B37] Khaligh-Razavi S.-M. Kriegeskorte N. (2014). Deep supervised, but not unsupervised, models may explain it cortical representation. PLoS Comput. Biol. 10:e1003915 10.1371/journal.pcbi.1003915 25375136 PMC4222664

[B38] Kriegeskorte N. Mur M. Ruff D. A. Kiani R. Bodurka J. Esteky H.. (2008). Matching categorical object representations in inferior temporal cortex of man and monkey. Neuron 60, 1126–1141. 10.1016/j.neuron.2008.10.043 19109916 PMC3143574

[B39] Krizhevsky A. Sutskever I. Hinton G. (2012). ImageNet classification with deep convolutional neural networks. Adv. Neural Inform. Process. Syst. 25, 1097–1105.

[B40] Lambon-Ralph M. Jefferies E. Patterson K. Rogers T. (2017). The neural and computational bases of semantic cognition. Nat. Rev. Neurosci. 18, 42–55. 10.1038/nrn.2016.150 27881854

[B41] Lescroart M. D. Biederman I. (2013). Cortical representation of medial axis structure. Cereb. Cortex 23, 629–637. 10.1093/cercor/bhs046 22387761

[B42] Liuzzi A. G. Bruffaerts R. Dupont P. Adamczuk K. Peeters R. De Deyne S.. (2015). Left perirhinal cortex codes for similarity in meaning between written words: comparison with auditory word input. Neuropsychologia 76, 4–16. 10.1016/j.neuropsychologia.2015.03.016 25795039

[B43] Liuzzi A. G. Bruffaerts R. Peeters R. Adamczuk K. Keuleers E. De Deyne S.. (2017). Cross-modal representation of spoken and written word meaning in left pars triangularis. Neuroimage 150, 292–307. 10.1016/j.neuroimage.2017.02.032 28213115

[B44] Lorenz S. Weiner K. S. Caspers J. Mohlberg H. Schleicher A. Bludau S.. (2017). Two new cytoarchitectonic areas on the human mid-fusiform gyrus. Cereb. Cortex (New York, N.Y. : 1991) 27, 373–385. 10.1093/cercor/bhv225 26464475 PMC6248695

[B45] Molenberghs P. Gillebert C. Peeters R. Vandenberghe R. (2008). Convergence between lesion-symptom mapping and fmri of spatially selective attention in the intact brain. J. Neurosci. 28, 3359–3373. 10.1523/JNEUROSCI.5247-07.2008 18367603 PMC6670586

[B46] Oldfield R. (1971). The assessment and analysis of handedness: the Edinburgh inventory. Neuropsychologia 9, 97–113. 10.1016/0028-3932(71)90067-4 5146491

[B47] Op de Beeck H. P. Torfs K. Wagemans J. (2008). Perceived shape similarity among unfamiliar objects and the organization of the human object vision pathway. J. Neurosci. 28, 10111–10123. 10.1523/JNEUROSCI.2511-08.2008 18829969 PMC6671279

[B48] Riesenhuber M. Poggio T. (2000). Models of object recognition. Nat. Neurosci. 3(Suppl.), 1199–1204. 10.1038/81479 11127838

[B49] Saygin A. Sereno M. (2008). Retinotopy and attention in human occipital, temporal, parietal, and frontal cortex. Cereb. Cortex 18, 2158–2168. 10.1093/cercor/bhm242 18234687

[B50] Scheperjans F. Eickhoff S. B. Hömke L. Mohlberg H. Hermann K. Amunts K.. (2008a). Probabilistic maps, morphometry, and variability of cytoarchitectonic areas in the human superior parietal cortex. Cereb. Cortex 18, 2141–2157. 10.1093/cercor/bhm241 18245042 PMC3140197

[B51] Scheperjans F. Hermann K. Eickhoff S. B. Amunts K. Schleicher A. Zilles K. (2008b). Observer-independent cytoarchitectonic mapping of the human superior parietal cortex. Cereb. Cortex 18, 846–867. 10.1093/cercor/bhm116 17644831

[B52] Scheperjans F. Palomero-Gallagher N. Grefkes C. Schleicher A. Zilles K. (2005). Transmitter receptors reveal segregation of cortical areas in the human superior parietal cortex: relations to visual and somatosensory regions. Neuroimage 28, 362–379. 10.1016/j.neuroimage.2005.06.028 16054841

[B53] Schrooten M. Ghumare E. G. Seynaeve L. Theys T. Dupont P. Van Paesschen W. (2017). Electrocorticography of spatial shifting and attentional selection in human superior parietal cortex. Front. Hum. Neurosci. 11:240 10.3389/fnhum.2017.00240 PMC5425472 28553217

[B54] Schwarzlose R. F. Swisher J. D. Dang S. Kanwisher N. (2008). The distribution of category and location information across object-selective regions in human visual cortex. Proc. Natl. Acad. Sci. U.S.A. 105, 4447–4452. 10.1073/pnas.0800431105 18326624 PMC2393746

[B55] Seghier M. (2013). The angular gyrus: multiple functions and multiple subdivisions. Neuroscientist 19, 43–61. 10.1177/1073858412440596 22547530 PMC4107834

[B56] Silver M. Ress D. Heeger D. (2005). Topographic maps of visual spatial attention in human parietal cortex. J. Neurophysiol. 94, 1358–1371. 10.1152/jn.01316.2004 15817643 PMC2367310

[B57] Silver M. A. Kastner S. (2009). Topographic maps in human frontal and parietal cortex. Trends Cogn. Sci. 13, 488–495. 10.1016/j.tics.2009.08.005 19758835 PMC2767426

[B58] Swisher J. Halko M. Merabet L. McMains S. Somers D. (2007). Visual topography of human intraparietal sulcus. J. Neurosci. 27, 5326–5337. 10.1523/JNEUROSCI.0991-07.2007 17507555 PMC6672354

[B59] Todd J. Marois R. (2004). Capacity limit of visual short-term memory in human posterior parietal cortex. Nature 428, 751–754. 10.1038/nature02466 15085133

[B60] Uddin L. Q. Supekar K. Amin H. Rykhlevskaia E. Nguyen D. A. Greicius M. D.. (2010). Dissociable connectivity within human angular gyrus and intraparietal sulcus: evidence from functional and structural connectivity. Cereb. Cortex 20, 2636–2646. 10.1093/cercor/bhq011 20154013 PMC2951845

[B61] Valyear K. F. Cavina-Pratesi C. Stiglick A. J. Culham J. C. (2007). Does tool-related fMRI activity within the intraparietal sulcus reflect the plan to grasp? NeuroImage 36(Suppl. 2), T94–T108. 10.1016/j.neuroimage.2007.03.031 17499175

[B62] Vandenberghe R. Geeraerts S. Molenberghs P. Lafosse C. Vandenbulcke M. Peeters K.. (2005). Attentional responses to unattended stimuli in human parietal cortex. Brain 128, 2843–2857. 10.1093/brain/awh522 15857928

[B63] Vandenberghe R. Gillebert C. R. (2009). Parcellation of parietal cortex: convergence between lesion-symptom mapping and mapping of the intact functioning brain. Behav. Brain Res. 199, 171–182. 10.1016/j.bbr.2008.12.005 19118580

[B64] Vandenberghe R. Gillebert C. R. (2013). Dissociations between spatial-attentional processes within parietal cortex: insights from hybrid spatial cueing and change detection paradigms. Front. Hum. Neurosci. 7:366. 10.3389/fnhum.2013.00366 23882202 PMC3712144

[B65] Vandenberghe R. Price C. Wise R. Josephs O. Frackowiak R. (1996). Functional anatomy of a common semantic system for words and pictures. Nature 383, 254–256. 10.1038/383254a0 8805700

[B66] Vandenberghe R. Wang Y. Nelissen N. Vandenbulcke M. Dhollander T. Sunaert S.. (2013). The associative-semantic network for words and pictures: effective connectivity and graph analysis. Brain Lang. 127, 264–272. 10.1016/j.bandl.2012.09.005 23084460

[B67] Vandenbulcke M. Peeters R. Fannes K. Vandenberghe R. (2006). Knowledge of visual attributes in the right hemisphere. Nat. Neurosci. 9, 964–970. 10.1038/nn1721 16767090

[B68] Vigneau M. Beaucousin V. Hervé P. Y. Duffau H. Crivello F. Houdé O.. (2006). Meta-analyzing left hemisphere language areas: phonology, semantics, and sentence processing. Neuroimage 30, 1414–1432. 10.1016/j.neuroimage.2005.11.002 16413796

[B69] Wojciulik E. Kanwisher N. (1999). The generality of parietal involvement in visual attention. Neuron 23, 747–764. 10.1016/S0896-6273(01)80033-7 10482241

[B70] Xu Y. Chun M. M. (2006). Dissociable neural mechanisms supporting visual short-term memory for objects. Nature 440, 91–95. 10.1038/nature04262 16382240

[B71] Xu Y. Lin Q. Han Z. He Y. Bi Y. (2016). Intrinsic functional network architecture of human semantic processing: modules and hubs. Neuroimage 132, 542–555. 10.1016/j.neuroimage.2016.03.004 26973170

[B72] Yantis S. Schwarzbach J. Serences J. Carlson R. Steinmetz M. Pekar J.. (2002). Transient neural activity in human parietal cortex during spatial attention shifts. Nat. Neurosci. 5, 995–1003. 10.1038/nn921 12219097

